# Comparative Genomic Analysis of N_2_-Fixing and Non-N_2_-Fixing *Paenibacillus* spp.: Organization, Evolution and Expression of the Nitrogen Fixation Genes

**DOI:** 10.1371/journal.pgen.1004231

**Published:** 2014-03-20

**Authors:** Jian-Bo Xie, Zhenglin Du, Lanqing Bai, Changfu Tian, Yunzhi Zhang, Jiu-Yan Xie, Tianshu Wang, Xiaomeng Liu, Xi Chen, Qi Cheng, Sanfeng Chen, Jilun Li

**Affiliations:** 1 Key Laboratory for Agrobiotechnology, China Agricultural University, Beijing, P. R. China; 2 Beijing Institute of Genomics, Chinese Academy of Sciences, Beijing, P. R. China; 3 Biotechnology Research Institute, Chinese Academy of Agricultural Sciences, Beijing, P. R. China; MicroTrek Incorporated, United States of America

## Abstract

We provide here a comparative genome analysis of 31 strains within the genus *Paenibacillus* including 11 new genomic sequences of N_2_-fixing strains. The heterogeneity of the 31 genomes (15 N_2_-fixing and 16 non-N_2_-fixing *Paenibacillus* strains) was reflected in the large size of the shell genome, which makes up approximately 65.2% of the genes in pan genome. Large numbers of transposable elements might be related to the heterogeneity. We discovered that a minimal and compact *nif* cluster comprising nine genes *nifB*, *nifH*, *nifD*, *nifK*, *nifE*, *nifN*, *nifX*, *hesA* and *nifV* encoding Mo-nitrogenase is conserved in the 15 N_2_-fixing strains. The *nif* cluster is under control of a σ^70^-depedent promoter and possesses a GlnR/TnrA-binding site in the promoter. Suf system encoding [Fe–S] cluster is highly conserved in N_2_-fixing and non-N_2_-fixing strains. Furthermore, we demonstrate that the *nif* cluster enabled *Escherichia coli* JM109 to fix nitrogen. Phylogeny of the concatenated NifHDK sequences indicates that *Paenibacillus* and *Frankia* are sister groups. Phylogeny of the concatenated 275 single-copy core genes suggests that the ancestral *Paenibacillus* did not fix nitrogen. The N_2_-fixing *Paenibacillus* strains were generated by acquiring the *nif* cluster via horizontal gene transfer (HGT) from a source related to *Frankia*. During the history of evolution, the *nif* cluster was lost, producing some non-N_2_-fixing strains, and *vnf* encoding V-nitrogenase or *anf* encoding Fe-nitrogenase was acquired, causing further diversification of some strains. In addition, some N_2_-fixing strains have additional *nif* and *nif*-like genes which may result from gene duplications. The evolution of nitrogen fixation in *Paenibacillus* involves a mix of gain, loss, HGT and duplication of *nif/anf/vnf* genes. This study not only reveals the organization and distribution of nitrogen fixation genes in *Paenibacillus*, but also provides insight into the complex evolutionary history of nitrogen fixation.

## Author Summary

We sequenced the genomes of 11 N_2_-fixing *Paenibacillus* strains and demonstrated the genomic diversity of the genus *Paenibacillus* by comparing these strains to each other and to 20 other strains (4 N_2_-fixing and 16 non-N_2_-fixing strains) that were sequenced previously. Phylogenetic analysis of the concatenated 275 single-copy core genes suggests that ancestral *Paenibacillus* did not fix nitrogen and the N_2_-fixing strains fall into two sub-groups, which were likely originated from a N_2_-fixing common ancestor. A minimal and compact *nif* cluster comprising nine *nif* genes encoding Mo-nitrogenase is highly conserved in the 15 N_2_-fixing strains. Variations in the *nif* cluster and in the chromosomal regions surrounding the *nif* cluster between two sub-groups imply at least two independent acquisitions with insertion of distinct *nif* cluster variants in different genomic sites of *Paenibacillus* in early evolutionary history. Phylogeny of the concatenated NifHDK sequences suggests that *Paenibacillus* and *Frankia* are sister groups. The *nif* cluster, a functional unit for nitrogen fixation, was lost, producing some non-N_2_-fixing strains. There were recent events of acquisition of *vnf* and *anf* genes, causing further diversification of some strains. The evolution of nitrogen fixation in *Paenibacillus* involves a mix of gain, loss, HGT and duplication of *nif/anf/vnf* genes.

## Introduction

Biological nitrogen fixation, the conversion of atmospheric N_2_ to NH_3_, plays an important role in the global nitrogen cycle and in world agriculture [1]. Nitrogen fixation is mainly catalyzed by the Mo-nitrogenase. The ability to fix nitrogen is widely, but sporadically distributed among Archaea and Bacteria which includes these families: Proteobacteria, Firmicutes, Cyanobacteria, Actinobacteria and Chlorobi [2]. Also, the contents and organization of nitrogen fixation (*nif*) genes vary significantly among the different N_2_-fixing organisms. For example, in *Klebsiella pneumoniae*, twenty *nif* genes are co-located within a ∼24 kb cluster [3], whereas in *Azotobacter vinelandii* the *nif* genes are more dispersed and distributed as two clusters in the genome [4]. The random distribution pattern and the difference in contents and organization of *nif* genes raise the question of origins and evolution of Mo-nitrogenase. Phylogenetic inference based on the sequences of *nif* genes is generally used to understand the evolution of *nif* genes [5]–[7]. Two conflicting hypotheses for origins of Mo-nitrogenase have been proposed on the basis of phylogenetic examination of Mo-nitrogenase protein sequences (NifHDK) [8]–[11]. One is the last common ancestor (LCA) hypothesis which implies that the Mo-nitrogenase had its origin in a common ancestor of the bacterial and archaeal domains. According to the LCA model gene loss has been extensive and accounts for the fact that nitrogenase is found neither in eukaryotes nor in many entire phyla of prokaryotes. The other is the methanogen origin hypothesis which implies that nitrogen fixation was originated in methanogenic archaea and subsequently was transferred into a primitive bacterium via horizontal gene transfer (HGT).

Remarkable progress in sequencing technology has advanced in understanding genetics and phylogenetic history of nitrogen fixation. For example, genome sequences of several diazotrophs, such as *Pseudomonas stutzeri* A1501 [12], *Herbaspirillum seropedicae* SmR1 [13] and *Wolinella succinogenes* [14], revealed that the Mo-nitrogenase genes constitute a nitrogen fixation cluster or island. The *nif* genes of *P. stutzeri*, including *nifQ*, *nifA*, *nifL*, *nifH*, *nifD*, *nifK*, *nifT*, *nifY*, *nifE*, *nifN*, *nifX*, *nifS*, *nifU*, *nifW*, *nifZ*, *nifM* and *nifF* are distributed in a 49-kb region. The *nif* genes of *H. seropedicae*, including *nifA*, *nifB*, *nifZ*, *nifZ1*, *nifH*, *nifD*, *nifK*, *nifE*, *nifN*, *nifX*, *nifQ*, *nifW*, *nifV*, *nifU* and *nifS* are in a region spanning 37 kb interspersed with *fix, mod, hes, fdx, hsc* and other genes. Variation of G+C content between the *nif* cluster and the genome average in *P. stutzeri* A1501 and existence of transposase near the *nif* cluster in *H. seropedicae* SmR1 are indicative of HGT of *nif* gene clusters [13]. However, since nitrogen fixation is an ancient complex process and is widely, but sporadically distributed among prokaryote families, further extensive genome sequences are needed to completely resolve the evolutionary history of nitrogenase.

Mo-nitrogenase is composed of two proteins, dinitrogenase (MoFe protein) and dinitrogenase reductase (Fe protein). The MoFe protein is an α_2_β_2_ heterotetramer (encoded by *nifDK*) that contains the iron–molybdenum cofactors (FeMo-co) and P clusters. The FeMo-co is a [Mo-7Fe-9S-homocitrate] cluster which serves as the active site of substrate binding and reduction. The P-cluster is a [8Fe-7S] cluster which shuttles electrons to the FeMo-co. The Fe protein is a γ_2_ homodimer (encoded by *nifH*) bridged by an intersubunit [4Fe-4S] cluster that serves as the obligate electron donor to the MoFe protein. In addition to the structural genes *nifHDK*, other genes *nifE nifN*, *nifX nifB*, *nifQ*, *nifV*, *nifY*, *nifU*, *nifS*, *nif*Z and *nifM* contribute to the synthesis of FeMo-co and maturation of nitrogenase [15]–[17]. Although the majority of present-day biological N_2_ reduction is catalyzed by the Mo-nitrogenase, two homologous alternative nitrogenases: V- and Fe-nitrogenase are important biological sources of fixed nitrogen in environments where Mo is limiting [18]. V- and Fe-nitrogenase are encoded by the *vnf* and *anf* genes. The Mo-, V- and Fe-nitrogenases are not equally distributed in nature. Most of diazotrophs, such as *K. pneumoniae*, possesses only the Mo-nitrogenase [19]. While some organisms, like *A. vinelandii*, possess all three types of nitrogenases [20] and other organisms, like *Rhodobacter capsulatus* and *Rhodospirillum rubrum*, carry the Mo- and Fe-nitrogenases [21], [22].

*Paenibacillus* is a large genus of Gram-positive, facultative anaerobic, endospore-forming bacteria. Members of this genus are biochemically and morphologically diverse and are found in various environments, such as soil, rhizosphere, insect larvae, and clinical samples [23]–[26]. Originally *Paenibacillus* was included within the genus *Bacillus*, however in 1993 it was reclassified as a separate genus [27]. At that time, the genus *Paenibacillus* encompassed 11 species including the three N_2_-fixing species *Paenibacillus polymyxa*, *Paenibacillus macerans* and *Paenibacillus azotofixans* [27]. The genus *Paenibacillus* currently comprises more than 120 named species, more than 20 of which have nitrogen fixation ability, including the following 8 novel species described by our laboratory: *Paenibacillus sabinae*, *Paenibacillus zanthoxyli*, *Paenibacillus forsythiae*, *Paenibacillus sonchi*, *Paenibacillus sophorae*, *Paenibacillus jilunlii*, *P. taohuashanense* and *P. beijingensis* [28]–[35]. Although diazotrophic *Paenibacillus* strains have potential uses as a bacterial fertilizer in agriculture, genomic information to date is limited and the genetics and evolution of nitrogen fixation of these diazotrophs are unknown.

Here we sequenced 11 N_2_-fixing *Paenibacillus* strains and compared these strains to each other and to 20 other strains (4 N_2_-fixing and 16 non-N_2_-fixing strains) that were sequenced previously. These strains were obtained from plant rhizhospheres, hot spring and human body and from Brazil, China, Korea, Israel, France, Belgium, United States of America, etc. (Table 1). Our study revealed that a *nif* gene cluster comprising *nifB*, *nifH*, *nifD*, *nifK*, *nifE*, *nifN*, *nifX*, *hesA* and *nifV* encoding Mo-nitrogenase is highly conserved in the 15 N_2_-fixing strains. Also, two homologous alternative nitrogenases: V- and Fe-nitrogenase encoded by the *vnf* and *anf* genes, respectively, are found in some *Paenibacillus* species. HGT, gene loss and gene duplication of *nif*, *vnf* and *anf* genes have contributed to evolution of nitrogen fixation in *Paenibacillus*. This study not only reveals the organization and distribution of *nif*/*anf/vnf* genes and the evolutionary patterns of nitrogen fixation in *Paenibacillus*, but also provides support for the methanogen origin hypothesis for *nif* evolution [10], [11].

**Table 1:** *Paenibacillus* strains used in study.

Strains	Source	Nitrogen fixer	Genome sequence
*Paenibacillus* sp. JDR2	Sweetgum stem wood, Florida, USA	−	[36]
*Paenibacillus* sp. Y412MC10	Obsidian hot spring, Montana, USA	−	[37]
*P. mucilaginosus* KNP414	Soil of Tianmu Mountain, Zhejiang, China	−	Unpublished
*P. mucilaginosus* K02	Soil of maize-farming fields, Guizhou, China	−	Unpublished
*P. mucilaginosus* 3016	Rhizosphere soil, Shandong, China	−	[38]
*P. polymyxa* E681	Rhizosphere of winter barley, Chonnam, South Korea	−	[39]
*P. polymyxa* SC2	Rhizosphere of pepper, Guizhou, China	−	[40]
*P. curdlanolyticus* YK9	Soil, Kobe city, Japan	−	Unpublished
*Paenibacillus* sp. HGF5	Human intestinal microflora, USA	−	Unpublished
*Paenibacillus* sp. HGF7	Human intestinal microflora, USA	−	Unpublished
*P. dendritiformis* C454	Soil, Tel Aviv, Israel	−	[41]
*P. elgii* B69	Soil samples, Hangzhou, China	−	[42]
*P. lactis* 154	Milk, Belgium	−	Unpublished
*P. peoriae* KCTC 3763	Soil, Republic of Korea	−	[43]
*Paenibacillus* sp. oral taxon786D14	Oral swab from female patient, USA	−	Unpublished
*P. vortex* V453	Rhizosphere, Tel Aviv, Israel	−	[44]
*P. polymyxa* WLY78	Bamboo rhizosphere, Beijing, China	*+*	Unpublished
*P. polymyxa* TD94	Scutellaria rhizosphere, Liaoning, China	*+*	This study
*P. polymyxa* 1–43	Corn rhizosphere, Shanxi, China	*+*	This study
*P.beijingensis* 1–18	Wheat rhizosphere, Beijing, China	*+*	This study
*Paenibacillus* sp. 1–49	Corn rhizosphere, Shanxi, China	*+*	This study
*Paenibacillus* sp. Aloe-11	Root of *Aloe chinensis*, Chongqing, China	+	[45]
*P. terrae* HPL-003	Soil of forest residue, Daejeon, Republic of Korea	+	[46]
*P. azotofixans* ATCC35681	Wheat roots, Parana state, Brazil	*+*	This study
*P. graminis* RSA19	Maize rhizosphere soil, Ramonville, France	*+*	This study
*P. sonchi* X19-5	Rhizosphere of Ku Caihua, Xinjiang, China	*+*	This study
*P. sophorae* S27	Rhizosphere of *Sophora japonica*, Beijing, China	*+*	This study
*P. massiliensis* T7	Willow rhizosphere, Beijing, China	*+*	This study
*P. zanthoxyli* JH29	Pepper rhizosphere, Hubei, China	*+*	This study
*P. forsythia* T98	Forsythia rhizosphere, Beijing, China	*+*	This study
*P. sabinae* T27	Rhizosphere of *Sabina squamata*, Beijing, China	*+*	Unpublished

## Results

### Genomic features

A summary of the features of each of the 11 newly-sequenced genomes of N_2_-fixing *Paenibacillus* strains and 20 previously-sequenced genomes of *Paenibacillus* strains (4 N_2_-fixers and 16 non-N_2_-fixers) is shown in Table 2. The characteristics (size, GC content, predicted number of coding sequences, and number of tRNA genes) of the 11 newly-sequenced genomes are within the range of previously-sequenced genomes of *Paenibacillus* strains (Table 2, Table S1). The 31 genomes vary in size by approximately three megabases (ranging from 4.90–8.77 Mb) with the number of CDSs ranging from 4460–9087, indicating substantial strain-to-strain variation. The G+C contents of the 31 genomes range from 44.2–58.4. The genome of *Paenibacillus sophorae* S27 has a larger size than those of the newly-sequenced strains.

**Table 2:** Genomic features of *Paenibacillus* strains.

Species	Status	GenBank accession number	Genome size (Mb)	G+C content	tRNA genes	Protein-coding sequences (CDSs)
*Paenibacillus* sp. JDR 2	Complete	CP001656.	7.18	50.3	87	6213
*Paenibacillus* sp. Y412MC10	Complete	CP001793	7.12	51.2	73	6238
*P.mucilaginosus* KNP414	Complete	CP002869	8.66	58.4	108	7811
*P. mucilaginosus* K02	Complete	CP003422	8.77	58.2	189	7252
*P. mucilaginosus* 3016	Complete	CP003235	8.74	58.3	170	7057
*P. polymyxa* E681	Complete	CP000154	5.39	45.8	91	4805
*P. polymyxa* SC2	Complete	CP002213	6.24	44.6	91	6032
*P. curdlanolyticus* YK9	Complete	AEDD00000000	5.45	51.9	101	4824
*Paenibacillus* sp. HGF5	Draft	AEXS00000000	6.95	51.0	71	6496
*Paenibacillus* sp. HGF7	Draft	AFDH00000000	6.28	52.8	72	5992
*P. dendritiformis* C454	Draft	AHKH00000000	6.38	54.0	31	5660
*P. elgii* B69	Draft	AFHW00000000	7.96	52.4	51	7777
*P. lactis* 154	Draft	AGIP00000000	6.81	51.8	74	6149
*P. peoriae* KCTC 3763	Draft	AGFX00000000	5.77	46.4	81	5073
*Paenibacillus* sp. oral taxon786 str. D14	Draft	ACIH00000000	4.90	51.8	69	4460
*P. vortex* V453	Draft	ADHJ00000000	6.39	48.8	57	5928
*P. polymyxa* WLY78	Draft	ALJV00000000	5.92	45.1	54	5729
*P. polymyxa* TD94	Draft	ASSA00000000	6.10	45.0	50	5697
*P. polymyxa* 1–43	Draft	ASRZ00000000	6.00	44.2	69	5731
*P. beijingensis* 1–18	Draft	ASSB00000000	5.44	46.0	59	5599
*Paenibacillus* sp. 1–49	Draft	ASRY00000000	5.65	46.4	56	5628
*Paenibacillus* sp. Aloe-11	Draft	AGFI00000000	5.79	46.6	73	5275
*P. terrae* HPL-003	Complete	CP003107	6.08	46.8	89	5525
*P. massiliensis* T7	Draft	ASSE00000000	6.32	48.4	63	5722
*P. graminis* RSA19	Draft	ASSG00000000	7.08	50.4	61	7081
*P. sonchi* X19-5	Draft	AJTY00000000	7.61	50.4	46	7705
*P.azotofixans* ATCC35681	Draft	ASQQ00000000	5.44	50.8	37	5924
*P. sophorae* S27	Draft	ASSF00000000	8.52	47.9	83	9087
*P. zanthoxyli* JH29	Draft	ASSD00000000	5.12	50.9	50	5622
*P. forsythia* T98	Draft	ASSC00000000	5.19	53.0	37	5552
*P. sabinae* T27	Complete	CP004078	5.27	52.6	82	5250

### Core and pan-genome analysis

**Figure 1:**
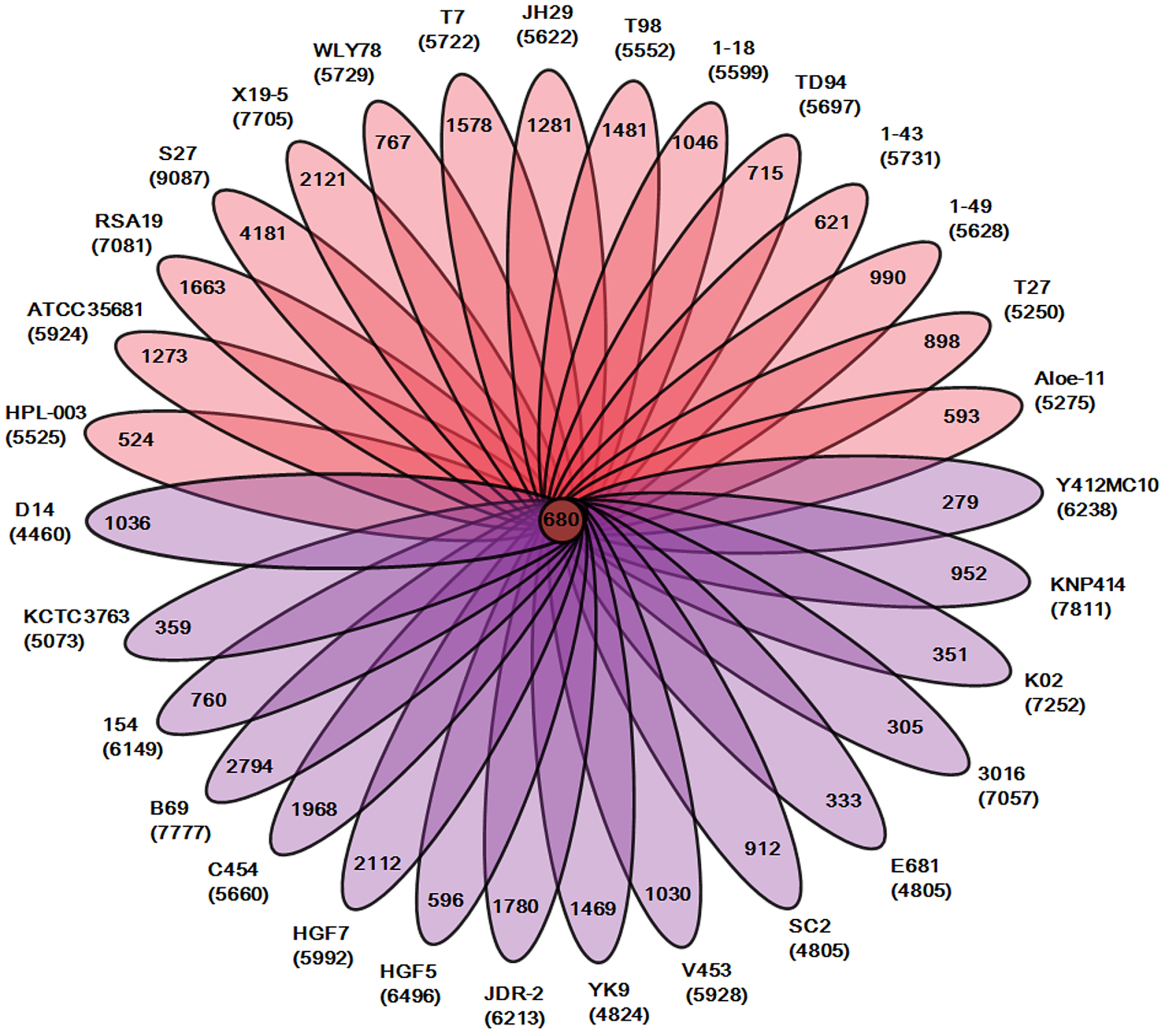
Genomic diversity of strains in the genus *Paenibacillus*. Each strain is represented by an oval that is colored: N_2_-fixing strains (red), non- N_2_-fixing strains (purple). The number of orthologous coding sequences (CDSs) shared by all strains (i.e., the core genome) is in the center. Overlapping regions show the number of CDSs conserved only within the specified genomes. Numbers in non-overlapping portions of each oval show the number of CDSs unique to each strain. The total number of protein coding genes within each genome is listed below the strain name.

Our analysis of the total 31 genomes reveals that a pan genome contains 55504 putative protein-coding genes in the genus *Paenibacillus*. Of the 55504 putative protein-coding genes, 37105, which made up 66.9% of the genes in the pan genome, were represented in only one genome of *Paenibacillus* spp., suggesting a high frequency of horizontal gene acquisition from other taxa. In contrast to the pan-genome, the genus *Paenibacillus* had the core genome of 680 putative protein-coding genes, which represents only 9% to 15% of the repertoire of protein coding genes of each strain, illustrating a large degree of genomic diversity in this group of bacteria (Figure 1). The genomic data are consistent with the fact that *Paenibacillus* strains are morphologically and physiologically diverse.

**Figure 2:**
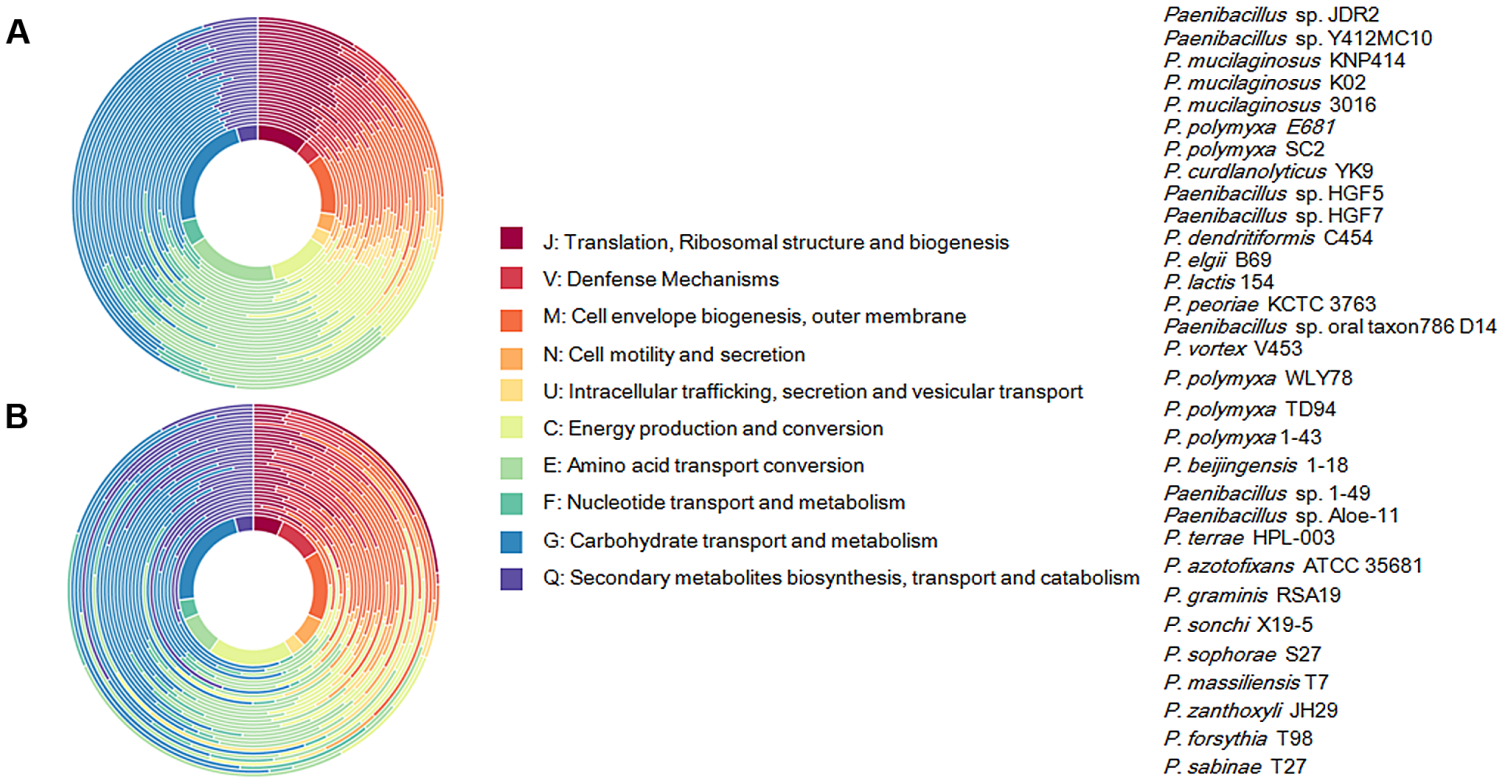
Functional classification of gene content of the 31 *Paenibacillus* strains. (A) Profiles of Cluster of Orthologous Groups (COG) showing percentage of genes in each category out of total annotated genes. Taxa from inside of circle to outside of circle are from *Paenibacillus* sp. JDR 2 (top in the strain list) to *P. sabinae* T27 (down in the strain list). (B) Profiles of COG showing function categories for genes in core genomes. Taxa from inside of circle to outside of circle are from *Paenibacillus* sp. JDR 2 (top in the strain list) to *P. sabinae* T27 (down in the strain list).

We further comparatively analyze the core genome of 15 N_2_-fixing and 16 non-N_2_-fixing *Paenibacillus* strains. We found that non-N_2_-fixing strains had the core genome of 908 putative protein-coding genes, which made up 12–20% of protein-coding genes in each strain. N_2_-fixing strains had the core genome of 1264 putative protein-coding genes, which code 14–24% of the protein pool in each genome. Further, we use Cluster of Orthologous Groups (COG) assignments to determine whether there were differences in the proportion of the core genome attributable to a particular cellular process (Figure 2 and Table S2). Interestingly, core genome of N_2_-fixing strains was found to be disproportionally enriched in cell motility and chemotaxis genes (Fisher's exact test; P value<0.01). Since these N_2_-fixing strains were isolated from plant rhizospheres, cell motility and chemotaxis are of importance for bacterial adaptation to the ever-changing rhizosphere environment [47].

### Transposable elements

In this study, transposons were identified using the ISfinder database (http://www-is.biotoul.fr/) and only expectation values of 10^−5^ and below were considered as significant matches during searches. Each *Paenibacillus* genome in this study contains a unique set of transposons (Table S3). The number of transposon copies pergenome ranges from 3 (*P. polymyxa* SC2) to 118 (*P. sophorae* S27). Members of the IS3, IS4, IS5, IS1182 and IS200/IS605 families are most common. However, there is not a large difference in numbers of transposable elements between other N_2_-fixing and non-N_2_-fixing strains.

### Prophage

Here prophages were identified using PHAST. Each genome of the 31 strains contains 1–10 prophages and/or prophage remnants, ranging in size from 14.4 to 59.1 kb. Collectively, the 31 genomes have 16 intact prophages and 69 prophage remnants. The newly-sequenced genomes have 38 prophages, most of which have a set of cargo genes that encode putative bacteriocins, DNA replication protein DnaD, ABC transporter ATP-binding protein, Non-ribosomal peptide synthase module containing protein adenine- and cytosine-specific DNA methyltransferases, and DNA/RNA helicase (Table S4). However, there is not a large difference in numbers of prophages between other N_2_-fixing and non-N_2_-fixing strains.

### The *nif* gene cluster is highly conserved in *Paenibacillus*

**Figure 3:**
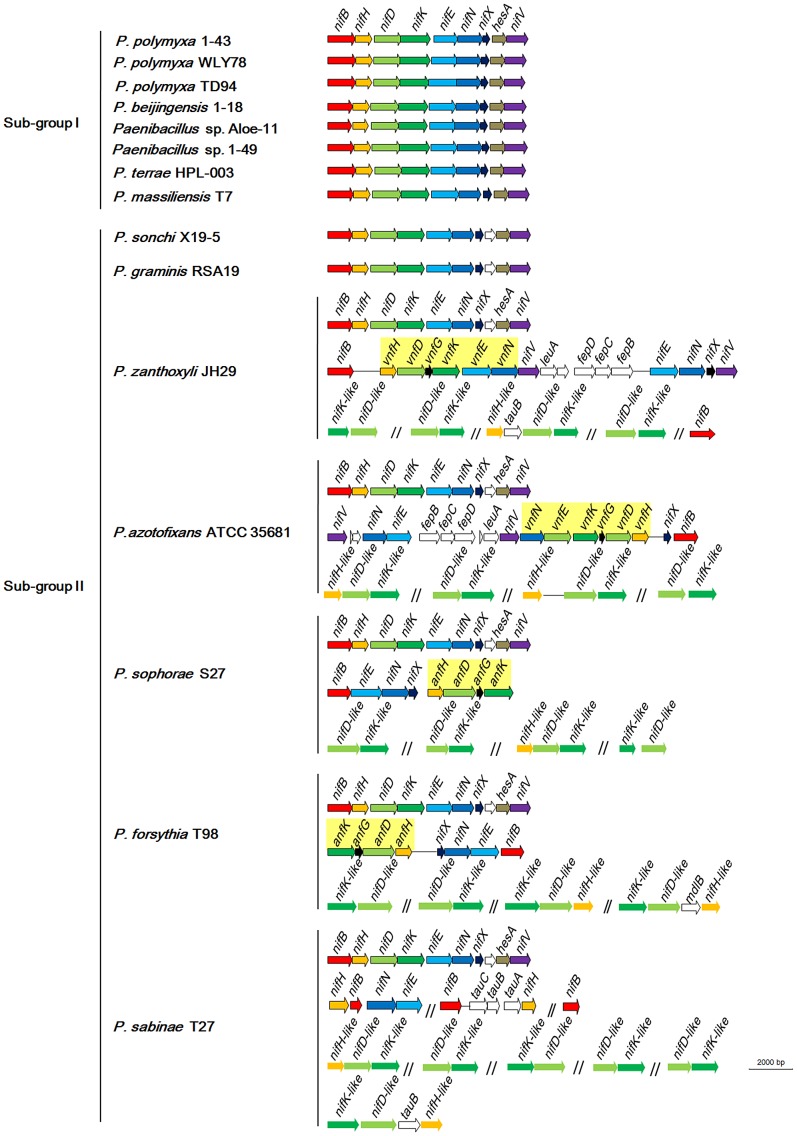
Organization of *nif*, *vnf*, *anf* and *nif*-like genes in N_2_-fixing *Paenibacillus* strains. *nif*, *vnf*, *anf* and *nif*-like genes are marked with different colors. The 9 *nif* genes *nifBHDKENXhesAnifV* are contiguous within Sub-group I and there is a *orf* between *nifX* and *hesA* within Sub-group II.

Comparison of COG assignments between non-N_2_-fixing and N_2_-fixing *Paenibacillus* strains (Table S2) revealed that 9 core genes in the N_2_-fixing strains: *nifB*, *nifH*, *nifD*, *nifK*, *nifE*, *nifN*, *nifX*, *hesA* and *nifV*, which are organized as a *nif* gene cluster arranged within an 10.5–12 kb genomic region, are conserved in all of the 15 N_2_-fixing strains (Figure 3, Table S5). The *nifH*, *nifD* and *nifK* are structural genes for Mo-nitrogenase, and the *nifB*, *nifE*, *nifN*, *nifX* and *nifV* are involved in synthesis of FeMo-cofactor. The gene *hesA*, which is located between *nifX* and *nifV*, is also found in the *nif* clusters of *Frankia* [48] and cyanobacteria [49]. HesA (also being called NAD/FAD-binding protein) is a member of the ThiF-MoeB-HesA family, which is involved in molybdopterin and thiamine biosynthesis. Our recent studies demonstrated that HesA is required for efficient nitrogen fixation in *Paenibacillus* [50]. As shown in Figure S1, the numbers of *nif* genes and size of the *nif* cluster of *Paenibacillus* are much smaller than those of *Frankia*, cyanobacteria, *Chlorobia* (green sulfur) and Proteobacteria.

**Figure 4:**
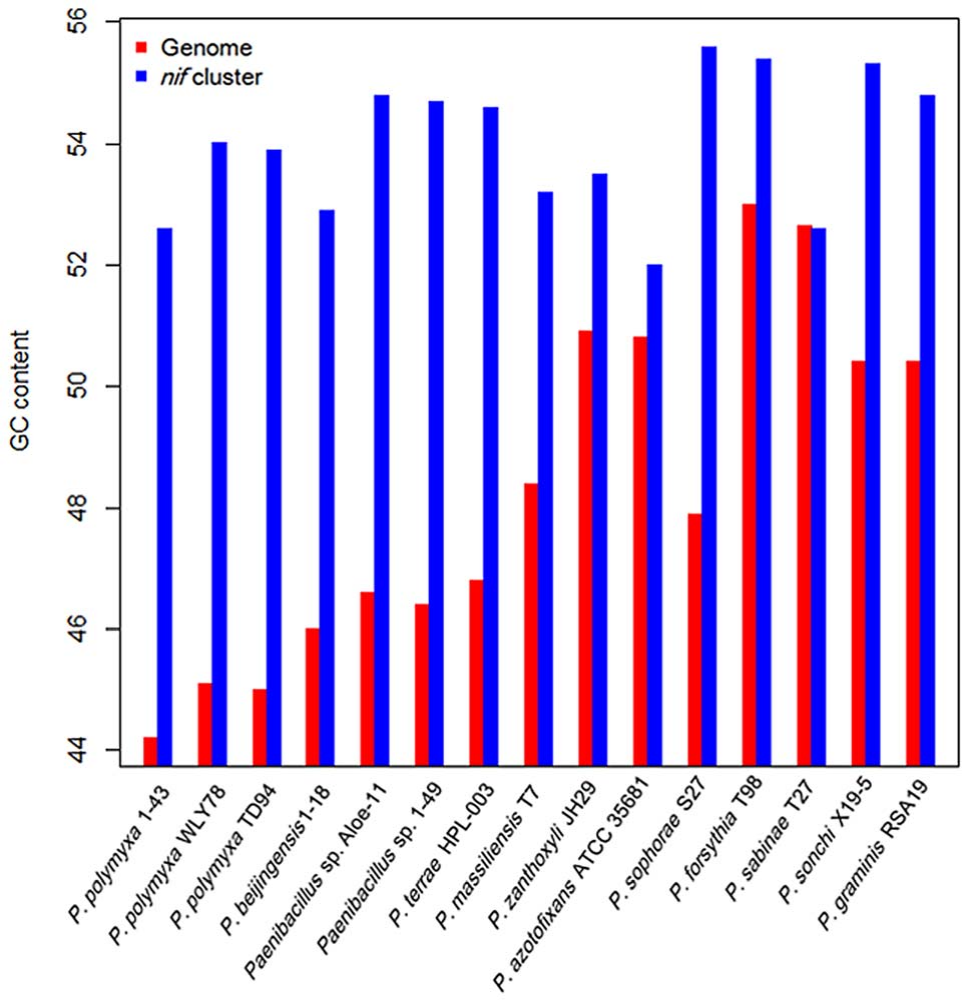
Comparison of G+C contents of the *nif* clusters with those of the average of the chromosomal genomes.

Although the *nif* gene cluster composed of *nifB*, *nifH*, *nifD*, *nifK*, *nifE*, *nifN*, *nifX*, *hesA* and *nifV* is highly conserved among the 15 N_2_-fixing *Paenibacillus* strains, there are some variations in DNA sequences of the *nif* clusters, which can be divided to two sub-groups: Sub-group I and Sub-group II. The 9 genes *nifBHDKENXhesAnifV* of the *nif* gene cluster within Sub-group I are contiguous, while there is an *orf* of 261–561 bp, whose predicted product is unknown, between *nifX* and *hesA* within Sub-group II. Except those of *P. massiliensis* T7 within Sub-group I, and *P. sonchi* X19-5 and *P. graminis* RSA19 within Sub-group II, the *nif* gene clusters generally exhibited more than 90% identity among each Sub-group and about 80% identity between two Sub-groups,

The G+C contents of the *nif* clusters are higher than those of the average of the entire genomes in other 14 N_2_-fixing *Paenibacillus* strains (52–55 vs. 44–54) except that the *nif* cluster of *P. sabinae* T27 has the same G+C with the genome (Figure 4). There is a transposase gene, an indicative of HGT, near the *nif* clusters of *Paenibacillus* sp. Aloe-11 and *P. sabinae* T27 (Figure S2). These data suggest that the *nif* clusters were acquired in *Paenibacillus* strains by HGT.

### Evolution of the *nif* gene cluster in *Paenibacillus*

**Figure 5:**
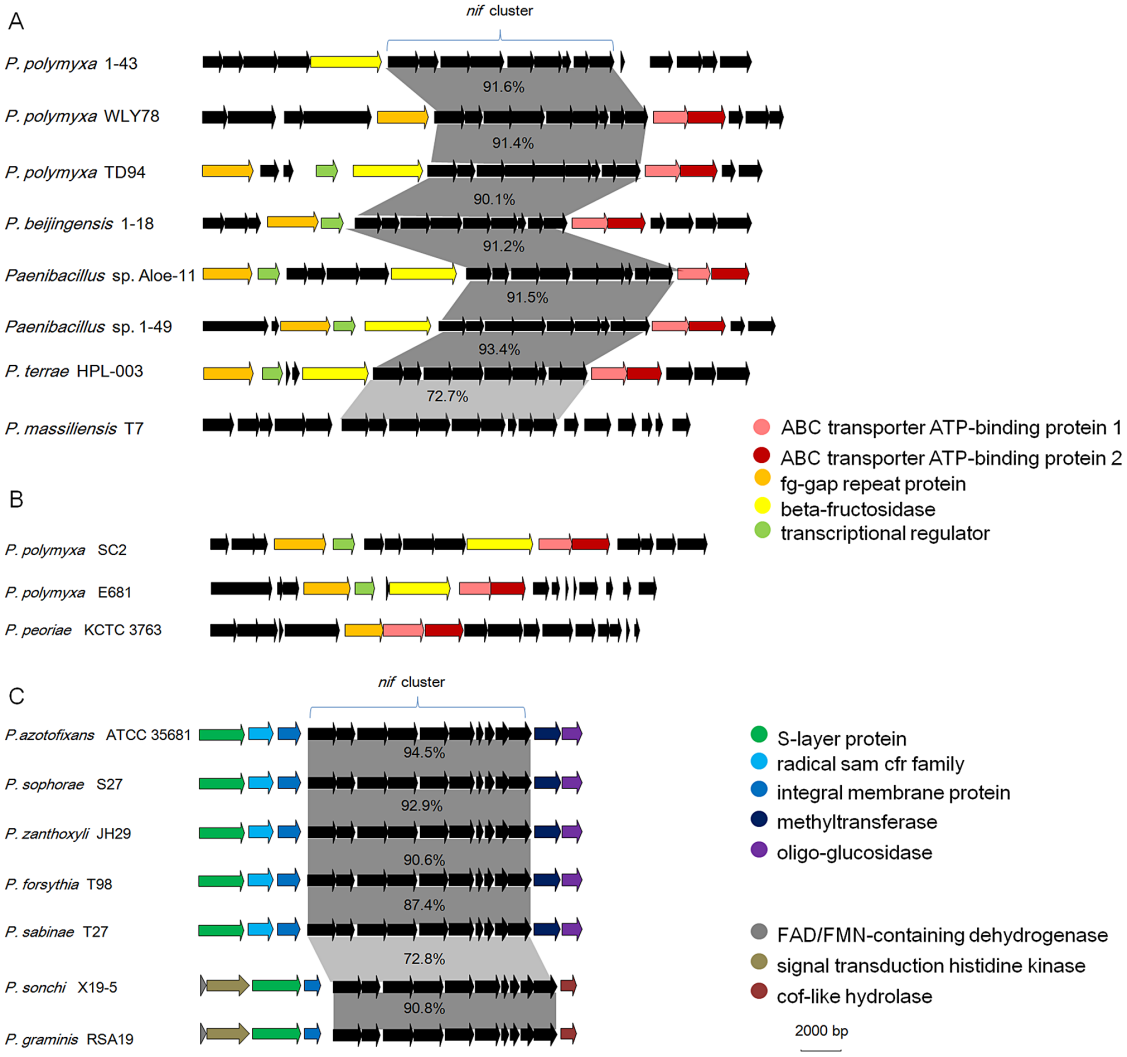
Synteny of the chromosomal regions flanking the *nif* gene cluster among each sub-group. (A) *nif* clusters of Sub-group I. (B) The chromosomal regions of non-N_2_-fixing strains corresponding to those flanking the *nif* gene cluster of Sub-group I. (C) *nif* clusters of Sub-group II.

To elucidate the evolution of the *nif* gene cluster in *Paenibacillus* strains, we further compared the chromosomal regions flanking the *nif* gene clusters to each other among the 15 N_2_-fixing *Paenibacillus* strains and to the corresponding chromosomal regions of the non-N_2_-fixing *Paenibacillus* strains. We found that ABC transporter ATP-binding protein gene and beta-fructosidase gene/fg-gap repeat protein gene were conserved in the downstream and upstream, respectively, of the *nif* clusters in the 7 N_2_-fixing *Paenibacillus* strains (*P. polymyxa* 1–43, *P. polymyxa* WLY78, *P. polymyxa* TD-94, *P. beijingensis* 1–18, *Paenibacillus*. sp. Aloe-11, *Paenibacillus* sp. 1–49 and *P. terrae* HPL-003) within Sub-group I (Figure 5A). Unlike in Sub-group I, integral membrane protein gene and FAD/FMN-containing dehydrogenase gene/methyltranferase gene were conserved in the downstream and upstream, respectively, of the *nif* clusters in all of the 7 N_2_-fixing *Paenibacillus* species (*P. sonchi* X19-5, *P. graminis* RSA19, *P. azotofixans* ATCC 35681, *P. sophorae* S27, *P. zanthoxyli* JH29, *P. forsythia* T98 and *P. sabinae* T27) within Sub-group II (Figure 5C). Combination of the findings that *nif* clusters fall into two sub-groups according to their identities, these data imply at least two independent acquisitions with insertion of distinct *nif* variants in different genomic sites of *Paenibacillus*.

Notably, the chromosomal regions flanking the *nif* gene clusters within Sub-group I are homologous to the corresponding regions of the non-N_2_-fixing *P. polmyxa* SC2, *P. polmyxa* E681 and *P. peoriae* KCTC 3763, suggesting that the *nif* cluster was lost in these strains (Figure 5B). Our results are consistent with the report that *nif* gene cluster was lost in cyanobacteria [49].

### Sporadic occurrence of alternative nitrogenase

As shown in Figure 3, in addition to the *nif* cluster encoding Mo-nitrogenase, 2 strains have *vnfHDGKEN* encoding V-nitrogenase and 2 strains have *anfHDGK* encoding Fe-nitrogenase. In *P. sophorae* S27 and *P. forsythia* T98, *anfHDGK* are linked with *nifBENX*, forming a 9.1–9.7 kb cluster. In *P. zanthoxyli* JH29 and *P. azotofixans* ATCC 35681, *vnfHDGKEN* are linked with *nifBENXV, fepBCD* (encoding iron-enterobactin transporter subunits), *leuA* and other unknown genes, forming a 20.4–20.9 kb cluster. These *anf/vnf* clusters are flanked by genes coding for hypothetical proteins. Each alternative nitrogenase cluster contains, as a minimum, *vnf*/*anfH*, *D*, *G*, and *K*. The organizations of *vnf* or *anf* are largely consistent, but distinct with those of *A. vinelandii* and *Methanococcus maripaludis* [4], [51]. It is most likely that *anf* or *vnf* gene cluster was recently horizontally transferred to N_2_-fixing strains which have already had a *nif* cluster, producing the *P. sophorae* S27, *P. forsythia* T98, *P. zanthoxyli* JH29 and *P. azotofixans*.

### The origin of *nif*/*vnf*/*anf* in *Paenibacillus*

**Figure 6:**
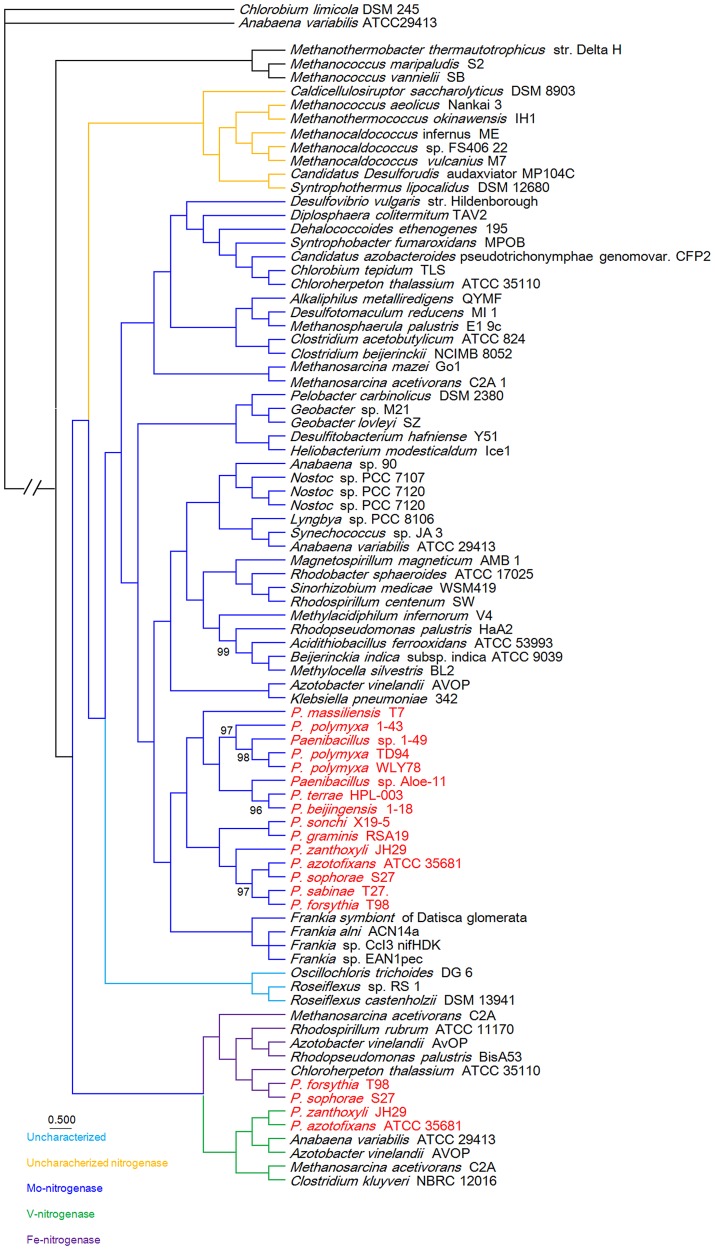
Bayesian inferred phylogenetic tree of concatenated NifHDK homologs. The interior node values of the tree are clade credibility values, values lower than 100% are indicated. Branches are colored blue (Mo-nitrogenase, Nif), green (V-nitrogenase, Vnf), purple (Fe-nitrogenase, Anf), light blue (uncharacterized homolog), dark yellow (uncharacterized nitrogenase). The text colored red was *Paenibacillus*.

**Figure 7:**
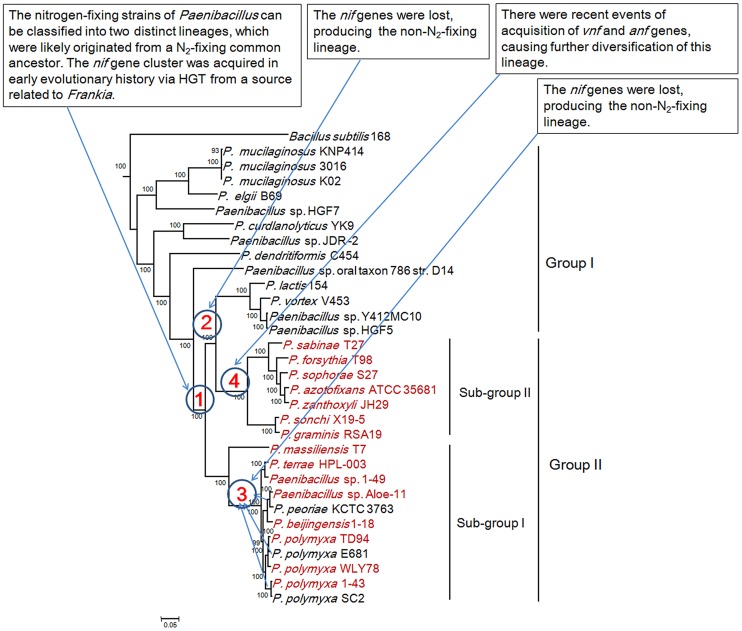
Maximum-likelihood phylogenetic tree of *Paenibacillus* strains and the 4 possible evolutionary pathways of nitrogen fixation in *Paenibacillus*. The tree was constructed based on 275 single-copy core proteins shared by the 31 *Paenibacillus* genomes and the rooting strain *B. subtilis* 168. Four likely pathways are marked with number 1–4.

To gain insights into the origin of *nif*/*vnf*/*anf* genes in *Paenibacillus*, a Bayesian inferred phylogenetic tree was constructed based on the concatenated Nif/Vnf/AnfHDK proteins. Results shown in Figure 6 indicate that Nif/Vnf/AnfHDK proteins of *Paenibacillus* strains fall into three distinct lineages. This phylogeny exhibits that NifHDK protein homologs formed two distinct clades, one of which was comprised of proteins from hydrogenotrophic methanogens and the other was comprised of proteins from both bacterial and methanogen genomes, in agreement with methanogen origin hypothesis of nitrogen fixation proposed by Boyd et al [10]. Our phylogenetic analysis of the concatenated NifHDK derived from the *nifHDK* of the *nif* clusters reveals that all of the 15 N_2_-fixing *Paenibacillus* strains form a coherent cluster consisting of two sub-groups, in agreement with the two sub-groups of *nif* clusters (Figure 7). Notably, the phylogeny reveals that *Paenibacillus* and *Frankia* are sister groups to the exclusion of the Firmicute *Clostridium*, implying that *Paenibacillus* and *Frankia* have a common *nif* gene ancestor. Phylogenies derived from each of the individual NifB, H, D, K, E, N, X and V are congruent with the phylogeny of the concatenated NifHDK (Figure S3, S4, S5, S6, S7, S8, S9, S10).

This phylogeny shows that Vnf/Anf proteins of *Paenibacillus* strains fall into the corresponding homologous lineages. Phylogeny derived from each of the individual VnfH/AnfH, D, G, K, E, N and X is congruent with the phylogeny of the concatenated Vnf/AnfHDK (Figure S3, S4, S5, S6, S7, S8, S9, S10). *anf* and *vnf* of *Paenibacillus* are nested with those of archaeon *M. acetivorans*, supporting that the ancestor of *anf* and *vnf* may originate from archaea.

### Phylogenetic analysis

We reconstructed the phylogeny of the 31 genomes based on the concatenation of the 275 core genes that are present in single copy in a genome. The 18 strains including 15 N_2_-fixing strains and 3 non-N_2_-fixing strains form a large group including two sub-groups and the other 13 non-N_2_-fixing strains fall into a large group (Figure 7). The clustering resulting from phylogenetic analysis corresponds well with the species assignments based on average nucleotide identity (ANI) using MUMmer (ANIm) (Table S6) [52]. For examples, *P. mucilaginosus* K02, *P. mucilaginosus* 3016 and *P. mucilaginosus* KNP414 have higher ANIm (98%). N_2_-fixing strains *P. polymyxa* 1–43, *P. polymyxa* WLY78 and *P. polymyxa* TD94 isolated from China, and non-N_2_-fixing strains *P. polymyxa* SC2 and *P. polymyxa* E681 isolated from China and South Korea, respectively, have higher ANIm (>95%). It is noteworthy that the other 2 unnamed strains Aloe-11 (ANIm≤87%) and 1–49 (ANIm<93%) may represent a novel species, respectively.

This phylogeny suggests that the *Paenibacillus* ancestor was probably non-fixing and the N_2_-fixing *Paenibacillus* strains appeared to occur much later than non-N_2_-fixing strains. Combination of the data that the *nif* cluster is conversed in the 15 N_2_-fixing *Paenibacillus* strains and the G+C contents of the *nif* clusters are higher than those of the average of the entire genomes, we proposed that N_2_-fixing *Paenibacillus* strains were generated by acquiring the *nif* cluster via HGT.

The N_2_-fixing strains of *Paenibacillus* fall into a large group composed of 2 distinct sub-groups (Sub-group I and Sub-group II), which were likely originated from a N_2_-fixing common ancestor. This species phylogeny is congruent with the phylogeny of *nif* genes. The phylogeny suggests that the 8 N_2_-fixing strains and the 3 non-N_2_-fixing strains within Sub-group I are most closely related. Nitrogen fixation may have been present in the ancestor of the 8 N_2_-fixing strains (*P. polymyxa* 1–43, *P. polymyxa* WLY78, *P. polymyxa* TD-94, *P. beijingensis*1–18, *Paenibacillus*. sp. Aloe-11, *Paenibacillus* sp. 1–49, *P. terrae* HPL-003 and *P. massiliensis* T7) and the 3 non-N_2_-fixing strains (*P. polymyxa* SC2, *P. polymyxa* E681 and *P. peoriae* KCTC 3763), and was later lost in the 3 non-N_2_-fixing strains. This phylogeny also shows that the 7 N_2_-fixing strains within Sub-group II (*P. sonchi* X19-5, *P. graminis* RSA19, *P. azotofixans* ATCC 35681, *P. sophorae* S27, *P. zanthoxyli* JH29, *P. forsythia* T98 and *P. sabinae* T27) are sister group with the 4 non-N_2_-fixing strains *P. lactis* 154, *P. vortex* V453, *Paenibacillus* sp. Y412MC10 and *Paenibacillus* sp. HGF5. Nitrogen fixation may have been present in the ancestor of the 7 N_2_-fixing and 4 non-N_2_-fixing strains and the *nif* genes were lost, producing the non-N_2_-fixing *P. lactis* 154 lineage.

Taken together, the *Paenibacillus* ancestor was probably non-fixing and the N_2_-fixing strains of *Paenibacillus* can be classified into 2 distinct sub-groups, which were likely originated from a N_2_-fixing common ancestor with minor variation in *nif* sequences. N_2_-fixing *Paenibacillus* strains were generated by acquiring the *nif* cluster in early evolutionary history via HGT from a source related to *Frankia*. After these initial acquisitions of the *nif* gene clusters, the strains that have them now have inherited them by vertical transmission. However, during the process of evolution, the *nif* cluster was lost, producing the 3 non-N_2_-fixing strains *P. polmyxa* SC2, *P. polmyxa* E681 and *P. peoriae* KCTC 3763 and the non-N_2_-fixing lineage *P. lactis* 154. There were recent events of acquisition of *vnf* and *anf* genes, causing further diversification of strains within Sub-group II. The most likely pathways of nitrogen fixation evolution are summarized in Figure 7.

### The *nif* gene cluster is a functional unit for nitrogen fixation

To investigate that the *nif* gene cluster is a functional unit for nitrogen fixation, the contiguous nine genes *nifBHDKENXhesAnifV* of the *nif* cluster and the *nifB* promoter from *P. beijinesis* 1–18, a representative of N_2_-fixing *Paenibacillus* strains, was PCR amplified and then constructed to vector pHY300PLK and further transferred to *E. coli* JM109. This yielded the recombinant *E. coli* strain 1–18. Nitrogenase activity was determined using the acetylene reduction assay (expressed as nmol C_2_H_4_/hr/mg protein) [53] and a ^15^N_2_ enrichment assay (expressed as δ^15^N) [54]. As shown in Figure S11, the nine genes *nifBHDKENXhesAnifV* within the *nif* cluster enabled *E. coli* to fix nitrogen, in agreement with our recent report obtained in *P. polmyxa* WLY78 [50]. The results indicate that the *nif* cluster is a functional unit for nitrogen fixation, and also a unit of HGT.

### The *nif* gene cluster possesses a σ^70^-dependent promoter and a GlnR/TnrA-binding site

We recently determined that the nine genes *nifB*, *nifH*, *nifD*, *nifK*, *nifE*, *nifN*, *nifX*, *hesA* and *nifV* within the *nif* gene cluster in *P. polmyxa* WLY78 were organized as an operon and that the *nifB* promoter of the *nif* cluster is a σ^70^-dependent promoter −35 (TTGACT) and −10 (TAAGAT) [50]. Here we revealed using bioinformatics analysis that the *nif* genes within the *nif* gene clusters among the other 14 N_2_-fixing *Paenibacillus* strains are organized as an operon and each of the *nif* clusters has a σ^70^-dependent promoter (Figure S12). The σ^70^-dependent promoter is very distinct from the typical σ^54^-dependent −24/−12 promoters found upstream of *nif* genes in Gram-negative N_2_-fixing bacteria, such as *K. pneumoniae* and *A. vinelandii*, whose *nif* gene expression requires the activation of the transcriptional activator NifA according to the concentration of ammonium and oxygen [55]. Although the σ^70^-dependent promoter is highly conserved among the 15 N_2_-fixing *Paenibacillus* strains, there are some variations in length of interval sequence between the putative transcriptional start site (TSS) and translation start codon (ATG) of *nifB* (Figure S12).

Unlike in Gram-negative diazotrophs, there is neither *nifA* gene encoding transcriptional activator NifA, nor NifA-binding site in the promoter region of the *nif* gene cluster. However, the genomes of the 15 N_2_-fixing *Paenibacillus* strains have *glnR* gene. In the Gram-positive model organism *Bacillus subtilis*, two transcriptional factors, TnrA and GlnR, control gene expression in response to nitrogen availability [56], [57]. TnrA activates and represses gene transcription when nitrogen is limiting for growth, while GlnR represses gene expression during growth with excess nitrogen. The two proteins bind to DNA sequences (GlnR/TnrA-sites) with a common consensus sequence (TGTNAN7TNACA) [56], [57]. Here we found that the GlnR/TnrA-binding sites exist in the *nif* promoter regions of the 15 N_2_-fixing *Paenibacillus* genomes (Figure S12). The GlnR/TnrA-binding sites are located upstream of the σ^70^-dependent promoter (−35 and −10) region in Sub-group I strains and some Sub-group II strains, while they are located downstream of the −35 and −10 regions in some Sub-group II strains. The existence of GlnR/TnrA-sites in *nif* promoter region suggests that regulation mechanisms of nitrogen fixation in *Paenibacillus* may be different from those of Gram-negative N_2_-fixing organisms.

### Suf system encoding [Fe–S] cluster is highly conserved in N_2_-fixing and non-N_2_-fixing *Paenibacillus* strains

Mo-nitrogenase is a complex [Fe-S] enzyme and the [Fe-S] clusters of nitrogenase play a critical function in electron transfer and in the reduction of substrates driven by the free energy liberated from Mg-ATP hydrolysis [19]. NifU and NifS are generally thought to be specialized for the nitrogenase [Fe-S] cluster assembly of nitrogen-fixing bacteria [58]. However, the genomes of the 15 N_2_-fixing *Paenibacillus* strains involved in this study do not possess homologues of *nifU* and *nifS*. Here we discovered that a Suf system (*sufCDSUB* operon) responsible for the formation of [Fe-S] clusters is highly conserved in N_2_-fixing and non-N_2_-fixing *Paenibacillus* strains. Suf system has been reported in *E. coli* (*sufABCDSE*) and some other organisms [59]. We deduce that *sufCDSUB* operon in N_2_-fixing *Paenibacillus* strains are involved in synthesis of the [Fe-S] clusters of nitrogenase and other FeS proteins. Perhaps it is because there is a *sufCDSUB* operon in non-N_2_-fixing *Paenibacillus* strain, a single event of HGT of the *nif* gene cluster will transfer a non-N_2_-fixing *Paenibacillus* strain to a N_2_-fixing *Paenibacillus* strain.

### Multiple *nif* genes in *Paenibacillus*

In addition to *nifBHDKENXhesAnifV* within the *nif* gene cluster, there is a set of additional *nifBEN* which are linked together with *vnf* or *anf* in the 4 species: *P. zanthoxyli* JH29 and *P. azotofixans* ATCC 35681, *P. sophorae* S27 and *P. forsythia* T98. Since the additional *nifBEN* form a cluster with *vnf* or *anf*, it is likely that they were horizontally transferred to the 4 species with *vnf* or *anf*. There are a cluster of *nifHBEN*, 2 *nifB* and 1 *nifH* located at different sites outside of the *nif* gene cluster in *P. sabinae* T27. The phylogenetic trees based on each of the individual NifB, NifH, NifE and NifN protein sequences (Figure S3, S4, S5, S6, S7, S8, S9, S10) show that each of them is clustered with its homolog derived from the *nif* gene clusters of *Paenibacillus*, suggesting that these genes derived from gene duplication. Transposases near the *nifBHEN* and *nifB* in *P. sabinae* T27 suggest that these genes may originate from gene duplication (Figure S2). Our previous results demonstrated that the 3 *nifH* genes from *P. sabinae* T27 could complement the *K. pneumoniae nifH^−^* mutant [60], suggesting that these *nifH* genes are functional in nitrogen fixation. However, we are not sure that the multiple *nifHBEN* are positively related to high nitrogenase activity.

### Multiple nitrogenase-like genes in *Paenibacillus*

**Figure 8:**
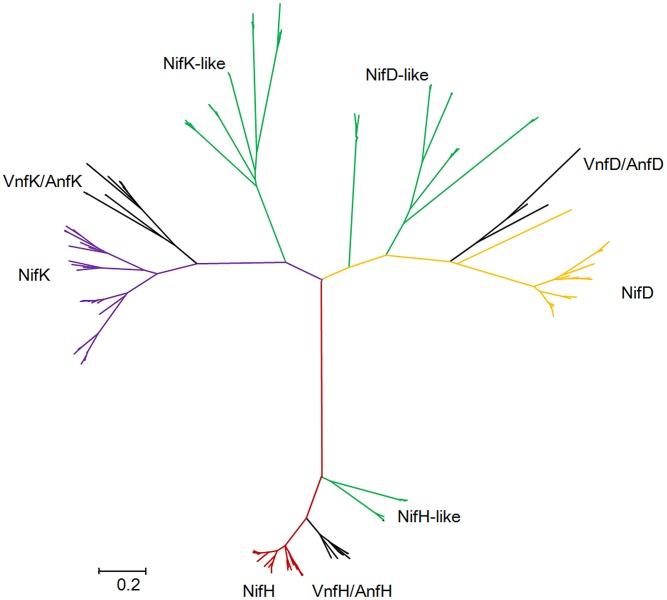
Maximum-likelihood phylogenetic tree of Mo-, Fe- and V-nitrogenases and nitrogenase-like sequences. Nif/Vnf/AnfH, Nif/Vnf/AnfD, Nif/Vnf/AnfK, Nif/Vnf/AnfH-like, Nif/Vnf/AnfD-like and Nif/Vnf/AnfK sequences were derived from the 15 N_2_-fixing *Paenibacillus* strains and other representative species.

Our studies revealed that there are nitrogenase-like genes including 1–2 *nifH*-like and 4–6 pairs of *nifDK*-like genes in the 5 species within Sub-group II: *P. azotofixans* ATCC 35681, *P. sophorae* S27, *P. zanthoxyli* JH29, *P. forsythia* T98 and *P. sabinae* T27 (Figure 3). Alignments of NifH-like sequences with NifH sequences show that 4Fe-4S iron sulfur cluster ligating cysteines (Cys97 and Cys132), ADP-ribose binding arginine (Arg101) and the P-loop/MgATP binding motif are invariant, suggesting that NifH-like proteins may function analogously to NifH (γ subunit of nitrogenase) (Figure S13). Conversely, NifD/NifK-like sequences are highly diverged from both α and β subunits of nitrogenase. For example, FeMoco-ligating residues at αCys275 and αHis442, and P-cluster-ligating residues at Cys62, Cys88 and Cys154 of NifD, are not conserved in NifD-like sequences (Figure S14). The residues ligating P-cluster at Cys70, Cys95 and Cys153 of NifK are not conserved in NifK-like sequences (Figure S15). Our results are in agreement with previous reports obtained in studies with Archaea and Firmicutes *Clostridium* [4], [8]. Further, phylogenetic analysis reveals that the NifH/NifD/NifK-like sequences form distinct groups which are clearly divergent from conventional nitrogenase (Figure 8).

## Discussion

In this study, we sequenced the genomes of 11 N_2_-fixing *Paenibacillus* strains and made a comparative genomic analysis with 20 other strains (4 N_2_-fixing and 16 non-N_2_-fixing strains) that were sequenced previously. Our analysis of the total 31 genomes revealed that of the 55504 putative protein-coding genes, 37105, which made up 66.9% of the genes in the pan genome, were represented in only one genome of *Paenibacillus* spp., suggesting a remarkable degree of HGT in shaping the genomes of each of the genus. It is generally accepted that abundance of mobile genetic elements correlates positively with the frequency of HGT. We discovered that each genome of all of the 31 strains contains 1–10 prophages and/or prophage remnants and 3–118 IS elements, supporting that these strains are rich in mobile genetic elements. The existence of transposable elements and prophage near the *nif* gene and *nif* gene cluster suggest that they may be involved in HGT and loss of *nif* genes. Our demonstration that the *nif* cluster from *P. beijinesis* 1–18 enabled *E. coli* to have nitrogen fixation ability supports that the *nif* cluster is a functional unit for nitrogen fixation and also a unit of HGT.

Genomic islands are known to have contributed to the evolution of microbial genomes by HGT in many bacteria, influencing traits such as antibiotic resistance, symbiosis and fitness, and adaptation in general [61]. The evolutionary advantage of genomic islands is that a large number of genes (e.g. operon, gene clusters encoding related functions) may be horizontally transferred and incorporated en bloc into the recipient genome in a single step [62]. Genome sequence analysis here revealed that nine genes *nifB*, *nifH*, *nifD*, *nifK*, *nifE*, *nifN*, *nifX*, *hesA* and *nifV* which are organized as a cluster arranged within 10.5–12 kb region are highly conserved in the 15 N_2_-fixing *Paenibacillus* strains. The sizes of *nif* clusters of *Paenibacillus* fall into the range of 10–200 kb genome islands in length. Also, the G+C contents of the *nif* clusters are higher than those of the average of the genomes in 14 N_2_-fixing strains except *P. sabinae* T27, in agreement with genome islands whose G+C content often differs from that of the rest of the genome. This favored the hypothesis that the *nif* region in *Paenibacillus* constitutes a nitrogen fixation island, as discovered in other nitrogen fixers [14], [63]. For example, *nif* genes are part of an island in *Wolinella succinogenes* [14] and in *Rhizobium leguminosarum* [63]. *nif* genes organized as clusters are also found in many other N_2_-fixing organisms. For examples, 20 *nif* genes are organized in 8 operons (*nifJC*, *nifHDKTY*, *nifEN*, *nifUSVW*, *nifZM*, *nifF*, *nifLA*, *nifBQ*) within ca. 24 kb of DNA in the chromosome of *K. pneumoniae* [3]. A total of 17–20 ORFs including 9–11 *nif* genes were organized as a cluster arranged within 17.3–18.5 kb regions among 4 *Frankia* strain: *Frankia* sp. EuIK1, *Frankia* sp. EAN1pec, *Frankia* sp. ACN14a and *Frankia* sp. HFPCcI3 [48]. In the *Cyanothece* 51142 genome, a representative of nonheterocystous cyanobacteria, the majority of genes involved in nitrogen fixation are located in a contiguous 28 kb cluster of 34 genes [49]. The different gene content and organization of *nif* genes indicate that complex evolutionary history of *nif* genes, and also suggest differences in protein requirements for nitrogenase synthesis and regulation of nitrogen fixation.

Phylogeny of the concatenated NifHDK proteins revealed that *Paenibacillus* and *Frankia* are sister groups to the exclusion of the Firmicute *Clostridium*, implying that *Paenibacillus* and *Frankia* have a common *nif* gene ancestor. Our results are consistent with the previous reports that *Frankia* and cyanobacterium *Anabaena* were sister groups to the exclusion of the Firmicute *Clostridium* [7]. Some common features found in the *nif* clusters support that *Paenibacillus* and *Frankia* are closely related. The first common feature is *hesA*, which is conserved in the *nif* clusters of *Paenibacillus, Frankia* and cyanobacteria, but not in N_2_-fixing Gram-negative and other Gram-positive bacteria, such as *Clostridium*. The second common feature is the compact organized *nifHDKENX* which is found in the *nif* clusters of *Paenibacillus* and *Frankia*, but not in *Clostridium* spp. In contrast, gene content and organization varied greatly between the *nif* clusters of *Paenibacillus* and *Clostridium*, although both genera *Paenibacillus* and *Clostridium* belong to the low G+C and Gram-positive Firmicutes. For example, *nifN-B* fusion gene was found in the *nif* gene clusters of the three species of *Clostridia*: *C. acetobutylicum*, *C. beijerinckii*, and *C. pasteurianum* [59], [64]. Also, the *nif* gene clusters of *C. acetobutylicum* and *C. beijerinckii* have *nifI1* and *nifI2* (homologs of *glnB*), which are involved in post-translational regulation of nitrogenase activity in response to fixed nitrogen [65]. These data suggest that the gene content and organization of the *nif* cluster of anaerobic *Clostridium* spp. are similar with those of *M. acetovorans* and *M. maripaudis* whose *nif* clusters also contain *nifI1* and *nifI2* located between *nifH* and *nifDK* [51], [65].

Phylogeny of the concatenated 275 single-copy core genes (Figure 7) suggests that the ancestral *Paenibacillus* did not fix nitrogen. Genome sequencing revealed that the *nif* cluster is highly conserved in all of the 15 N_2_-fixing strains and the G+C contents of the *nif* clusters are higher than those of the average of the genomes in 14 N_2_-fixing strains except *P. sabinae* T27. Also, phylogeny of the concatenated NifHDK proteins (Figure 6) revealed that *Paenibacillus* and *Frankia* are sister groups. All of these facts and evidences indicate that N_2_-fixing *Paenibacillus* strains may be generated by acquiring the *nif* cluster via HGT from a source related to *Frankia* in early evolutionary history. Strain phylogeny (Figure 7) also shows that the 15 N_2_-fixing strains of *Paenibacillus* fall into 2 distinct sub-groups, consistent with phylogeny of *nif* genes (Figure 6). The *nif* clusters show some variation between two sub-groups, and the genes surrounding the *nif* clusters from two Sub-groups are conserved and distinct. These data imply at least two independent acquisitions with insertion of distinct *nif* variants in different genomic sites of *Paenibacillus*.

Furthermore, strain phylogeny suggests that nitrogen fixation may have been present in the ancestor of the 8 N_2_-fixing strains (*P. polymyxa* 1–43, *P. polymyxa* WLY78, *P. polymyxa* TD94, *P. beijingensis*1–18, *Paenibacillus*. sp. Aloe-11, *Paenibacillus* sp. 1–49, *P. terrae* HPL-003 and *P. massiliensis* T7) and the 3 non-N_2_-fixing strains (*P. polmyxa* SC2, *P. polmyxa* E681 and *P. peoriae*KCTC 3763) within Sub-group I, and was later lost in the 3 non-N_2_-fixing strains (*P. polmyxa* SC2, *P. polmyxa* E681 and *P. peoriae* KCTC 3763). Notably, the model *P. polymyxa* is a N_2_-fixing species, and now this species includes both N_2_-fixing and non-N_2_-fixing strains. These closely related strains of this group were isolated from plant rhizospheres and from different geological locations of China, South Korea and Republic of Korea. Likewise, it is likely that nitrogen fixation may have been present and was later lost in the non-N_2_-fixing lineage *P. lactis* 154. The members of this lineage were isolated from complex locations. For examples, *P. lactis* 154 was isolated from milk, *Paenibacillus* sp. HGF5 from human intestinal microflora and *Paenibacillus* sp. Y412MC10 from hot spring, and *P. vortex* V453 is known to develop complex colonies with intricate architectures.

The newly sequenced genomes revealed that the 4 *Paenibacillus* species *P. sophorae* S27, *P. forsythia* T98, *P. zanthoxyli* JH29 and *P. azotofixans* have the second *nif* cluster which carrying *vnf* or *anf*, in addition to the *nif* cluster. *anfHDGK* are clustered with *nifBENX* in a 9.1–9.7 kb region in *P. sophorae* S27 and *P. forsythia* T98, *vnfHDGKEN* are clustered with *nifBENXV, fepBCD, leuA* and other unknown genes in a 20.4–20.9 kb region in *P. zanthoxyli* JH29 and *P. azotofixans* ATCC 35681. Phylogeny of the concatenated Nif/Anf/VnfHDK proteins indicates that *anfHDGK* and *vnfHDGKEN* of *Paenibacillus* originate differently from *nifHDK*, and may be not duplicated from their *nifHDK*. It is most likely that the *nif* cluster carrying *anf/vnf genes* was recently horizontally transferred to N_2_-fixing strains which have already had the *nif* cluster, producing *P. sophorae* S27, *P. forsythia* T98, *P. zanthoxyli* JH29 and *P. azotofixans*. These species were isolated from plant rhizosphere from China and Brazil. Our results are consistent with the recent reports that both Nif and Anf evolved in the methanogenic archaea, and *anf* or *vnf* derived from duplication of *nif* [8]. As described above, phylogenies of the concatenated Anf/VnfHDK and each of individual Anf/VnfH, D and K show that *Paenibacillus* strains fall into Anf and Vnf clusters, respectively. However, we found that the conserved residues in the P-loop binding motif of AnfH do not exist in *P. sophorae* S27, and the residues ligating P-cluster at Cys70 and Cys95 of VnfK do not exist in *P. zanthoxyli* JH29. Perhaps the residues ligating P-cluster or in P-loop binding motif are located on the other sites in VnfK and AnfH, respectively.

This study reveals that HGT of *nif/anf/vnf* gene cluster contributed to evolution of nitrogen fixation in *Paenibacillus*. Usually, a vehicle is needed to transfer genes efficiently between different species. It is thought that foreign DNAs are mainly transferred by means of plasmids or bacteriophages, as well as direct uptake by the host itself [58], [66], [67]. The best studied example of HGT of *nif* genes is symbiosis island of *Mesorhizobium loti*. The symbiosis island, a 502-kb chromosomally integrated element containing *nif* genes, was integrated into a phenylalanine tRNA gene mediated by a P4-type integrase encoded at the left end of the symbiosis island [68]–[70]. However, a phenylalanine tRNA gene near the *nif* cluster is not found, suggesting that it may be not transferred by P4-type integrase. But we found that there is a transposase gene, an indicative of HGT, near the *nif* clusters of *Paenibacillus* sp. Aloe-11 and *P. sabinae* T27 and near the *anf* cluster of *P. sophorae* S27. Also, a transcriptional regulator gene of *araC* type, which is known to be involved primarily in regulating pathogenicity islands in some bacteria but is also present in nonpathogenic organisms [62], neighbors the *nif* clusters of *P. polmyxa* TD94 and *Paenibacillus* sp. 1–11.

The deviant G+C content is one of the indicative used to detect HGT [67]. The G+C contents of the *nif* clusters are higher than those of the average of the entire genomes (52–55 vs. 44–53) in the 14 N_2_-fixing *Paenibacillus* strains except *P. sabinae* T27, supporting that the *nif* gene clusters in these strains are acquired by HGT. The similar G+C contents and high identities of *nif* genes among the 15 *nif* clusters suggest that these *nif* clusters originated from a common ancestor with minor variation. The G+C contents of the *anf* cluster is higher than the average of the genome in *P. sophorae* S27 (51% vs. 40%), and is lower than the average of the genome in *P. forsythia* T98 (51% vs. 53%). The G+C contents of the *vnf* cluster is the same (51% vs. 51%) as the average of the chromosomal genome in *P. azotofixans* ATCC 35681 and *P. zanthoxyli* JH29. A higher G+C contents of the *nif* cluster were found in some N_2_-fixing bacteria, such as *P. stutzeri* A1501 (66.8% vs. 63.8%) [12]. In rhizobia, the *nif* genes are located on either plasmids or genomic islands, which are prone to transfer between related bacteria [71]. However, the G+C contents of these plasmids and genomic islands are generally lower than the average of the chromosomal genome [72]–[74]. However, the G+C contents of the *nif* clusters are similar with those of the average of the entire genomes in the sequenced *Frankia* strains (69% vs. 70% in *Frankia* sp. HFPCcI3, 70% vs. 71% in *Frankia* sp. EAN1pec and 71% vs. 72% in *Frankia alni* ACN14a). It is generally accepted that although the deviant G+C content can be used to detect HGT, detection of HGT depends on a combination of several methods. This is because it is hard to detect HGT via deviant G+C content, if HGT occurred between the organisms with the same G+C contents [67].

Our genome sequencing revealed that there are nitrogenase-like genes including 1–2 *nifH*-like and 4–6 pairs of *nifDK*-like genes in the 5 species within Sub-group II: *P. azotofixans* ATCC 35681, *P. sophorae* S27, *P. zanthoxyli* JH29, *P. forsythia* T98 and *P. sabinae* T27 (Figure 3 and Table S5). Alignment of conserved residues ligating 4Fe-4S in NifH and ligating P-cluster and FeMoco and phylogenetic analysis in in NifD/K revealed that the *nif*-like and *nifDK*-like genes are clustered with those of archaea and Firmicutes such as Clostridia [4]. The data that NifH/NifD/NifK-like sequences fall into distinct groups by phylogenetic analysis suggest that multiple *nifH*-like and *nifDK*-like genes may result from gene duplication. The existence of transposases near the *nifDK*-like genes also suggested that multiple *nifDK*-like genes may result from gene duplication. It was proposed that Nif emerged from a nitrogenase-like ancestor approximately 1.5–2.2 Ga [10]. We wonder why there are so many *nifDK*-like genes in these *Paenibacillus* species. The determination of the function of nitrogenase-like genes will clarify their relation with nitrogen fixation.

## Materials and Methods

### Genome sequencing, assembly, and annotation

The draft sequences of 11 test *Paenibacillus* strains were produced by using Illumina paired-end sequencing technology at the BGI–Shenzhen (Table 2). Assembly was conducted by using SOAPdenovo v. 1.04 assembler [75]. Gene prediction was made using Glimmer v3.0 [76]. Annotation of protein coding sequence was performed by using the Basic Local Alignment Search Tool (BLAST) against the COG, Kyoto Encyclopedia of Genes and Genomes (KEGG) databases and NCBI nr protein database. The draft genomes of the 11 test *Paenibacillus* strains have been deposited in GenBank and the project accession numbers are listed in Table 2. Prophage was identified using PHAST [77].

### Comparative genomics

Pan Genome Analysis Pipeline of PGAP [78] was used to identify all of the orthologous pairs between test *Paenibacillus* genomes. The common dataset of shared genes among test strains was defined as their core genome. The total set of genes within test genomes was defined as the pan genome. The set of genes in each strain not shared with other strains was defined as unique genes. The average nucleotide identity (ANI) between strains of the 31 sequenced genomes were calculated using MUMmer [52]. Multiple alignment of conserved genomic sequence was using Mauve [79]. The genomes sequenced in this study are listed in Table S1.

### Phylogenetic analysis

Single gene alignments were aligned with molecular evolutionary genetics analysis (MEGA) [80]. The neighbor-joining trees were constructed by using the same software, and 1,000 bootstraps were done. Bayesian inferred phylogenetic tree of concatenated HDK homologs was generated using the MrBayes package [81]. A maximum-likelihood phylogenetic tree of *Paenibacillus* species was constructed based on 275 single-copy core proteins shared by 31 *Paenibacillus* genomes and the genome of *Bacillus subtilis* 168 according to the following methods: (i) multiple alignment of amino acid sequences were carried out by ClustalW (version 2.1) [82] (ii) conserved blocks from multiple alignment of test protein were selected by using Gblocks [83] (iii) ML tree were constructed using PhyML (version 3.0) [84] software (iv) CONSEL program [85] was used to select the best model of the trees.

### Construction of the recombinant plasmid and *E. coli* strain

Genomic DNA of diazotrophic *P. beijingensis* 1–18 was used as a template for cloning *nif* genes. A 10.7 kb Xba I -BamH I DNA fragment containing the *nif* cluster (a 300 bp promoter region and the contiguous nine genes *nifBHDKENXhesAnifV* and 184 bp downstream of the stop codon TAA of *nifV*) was PCR amplified with primers *nif* cluster-up (5′-TGCTCTAGAGGGAATATAACGTGGAGAGG-3′) and *nif* cluster-down (5′-CGCGGATCCCATTATACAGCACTATATTG-3′) and then ligated to Xba I and BamH I sites of pHY300PLK, yielding plasmid pHY300-18 (P*nif*+*nif* cluster). The plasmid was then transferred to *E. coli* JM109, yielding the recombinant *E. coli* 1–18.

### Acetylene reduction assays

For acetylene reduction assays, *P. beijingensis* 1–18 and the recombinant *E. coli* strain 18 were grown overnight in LD medium, then diluted into nitrogen-deficient medium and grown for 15–18 h. Following this stage, the cultures were collected and resuspended in an N-free medium to an OD_600_ of 0.2–0.4 in a serum bottle for nitrogenase derepression. The serum bottle was vacuumed and charged with argon gas. After 5–6 h, C_2_H_2_ (10% of the headspace volume) was injected into the serum bottle. After 30 min to 1 h, C_2_H_4_ was analyzed by Gas Chromatography [53].

### ^15^N_2_ incorporation assay

*Paenibacillus*sp.1–18 and the recombinant *E. coli* strain 1–18 were grown overnight in LD medium. The cultures were collected and resuspended in 70 ml N-free medium to an OD_600_ of 0.4 in the 120 ml serum bottle. The serum bottles were filled with N_2_ gas, and then 8-ml gas was removed and 5 ml ^15^N_2_ (99%+, Shanghai Engineering Research Center for Stable Isotope) gas was injected. After 72 hours of incubation at 30°C, the cultures were collected, freeze dried, ground, weighed and sealed into tin capsules. Isotope ratios are expressed as δ^15^N whose values are a linear transform of the isotope ratios ^15^N/^14^N, representing the per mille difference between the isotope ratios in a sample and in the atmospheric N_2_ [54].

### Data access

The genome sequences used in this study were submitted to the GenBank, the accession number was shown in Table 2.

## Supporting Information

Figure S1 Comparison of the *nif* gene cluster of *Paenibacillus* with those of the representative N_2_-fixing bacteria and archaea. (A) *Paenibacillus polymyxa* 1–43, (B) *Azotobacter vinelandii*, (C) *Klebsiella oxytoca* M5al, (D) *Nostoc punctiforme* PCC 73102, (E) *Frankia* sp. EAN1pec, (F) *Clostridium acetobutylicum*, (G) *Methanococus maripaludis*.
(TIF)

Figure S2 IS elements or prophages linked with the *nif* gene, *nif* cluster and *nif*-like genes.
(TIF)

Figure S3 Neighbor joining phylogenetic tree of the NifB sequences derived from *Paenibacillus* and other representative species. A total of 1,000 bootstrap replicates were made, and bootstrap values are indicated at each node.
(TIF)

Figure S4 Neighbor joining phylogenetic tree of the NifH, VnfH, AnfH and NifH-like protein sequences derived from *Paenibacillus* and other representative species. A total of 1,000 bootstrap replicates were made, and bootstrap values are indicated at each node.
(TIF)

Figure S5 Neighbor joining phylogenetic tree of the NifD, VnfD, AnfD and NifD-like protein sequences derived from *Paenibacillus* and other representative species. A total of 1,000 bootstrap replicates were made, and bootstrap values are indicated at each node.
(TIF)

Figure S6 Neighbor joining phylogenetic tree of the NifK, VnfK, AnfK and NifK-like protein sequences derived from *Paenibacillus* and other representative species. A total of 1,000 bootstrap replicates were made, and bootstrap values are indicated at each node.
(TIF)

Figure S7 Neighbor joining phylogenetic tree of the NifE, VnfE and NifE-like protein sequences derived from *Paenibacillus* and other representative species. A total of 1,000 bootstrap replicates were made, and bootstrap values are indicated at each node.
(TIF)

Figure S8 Neighbor joining phylogenetic tree of the NifN, VnfN and NifN-like protein sequences derived from *Paenibacillus* and other representative species. A total of 1,000 bootstrap replicates were made, and bootstrap values are indicated at each node.
(TIF)

Figure S9 Neighbor joining phylogenetic tree of the NifX protein sequences derived from *Paenibacillus* and other representative species. A total of 1,000 bootstrap replicates were made, and bootstrap values are indicated at each node.
(TIF)

Figure S10 Neighbor joining phylogenetic tree of the NifV protein sequences derived from *Paenibacillus* and other representative species. A total of 1,000 bootstrap replicates were made, and bootstrap values are indicated at each node.
(TIF)

Figure S11 Nitrogen fixation abilities of *P. beijingensis* 1–18 (WT) and recombinant *E. coli* 1–18 strain. (A) Nitrogenase activities determined by using acetylene reduction assay. (B) Nitrogen fixation ability determined by using for ^15^N_2_ incorporation. Error bars indicate the standard deviation observed from at least two independent experiments.
(TIF)

Figure S12 The σ^70^-depedent promoters of the *nif* clusters and the GlnR/TnrA-binding sites in the *nif* promoter regions in *Paenibacillus* strains.
(TIF)

Figure S13 Alignments of crucial residues surrounding the P-loop/MgATP binding motif, cysteine ligating 4Fe-4S and arginine ligating ADP-ribose in NifH and NifH-like protein sequences from *Paenibacillus* and other organisms.
(TIF)

Figure S14 Alignments of crucial residues ligating FeMo-co or P-cluster in NifD and NifD-like protein sequences from *Paenibacillus* and other organisms.
(TIF)

Figure S15 Alignments of crucial residues ligating FeMo-co or P-cluster in in NifK and NifK-like protein sequences from *Paenibacillus* and other organisms.
(TIF)

Table S1 The genomes sequenced in this study.
(DOCX)

Table S2 Comparison of COG assignments between non-N_2_-fixing and N_2_-fixing *Paenibacillus* strains.
(DOCX)

Table S3 Transposons present in the genomes of 31 *Paenibacillus* strains. The following information is provided for each putative transposon in genomes: transposon family, transposases, and numbers of copies of intact or remnant transposons in each genome.
(DOCX)

Table S4 Prophages present in the genomes of 31 *Paenibacillus* strains. The following information is provided for each prophage: insertion site, size, locus tags, and selected cargo genes.
(DOCX)

Table S5 The nitrogen fixation genes and nitrogenase-like genes in the nitrogen-fixing *Paenibacillus* strains.
(DOCX)

Table S6 Average Nucleotide Identity (%) based on whole genome alignments.
(XLSX)
