# Supplementary figures and images for: Comparative Genomic Analysis of N2-Fixing and Non-N2-Fixing Paenibacillus spp.: Organization, Evolution and Expression of the Nitrogen Fixation Genes
Source: PLoS Genet. 2014 Mar 20;10(3):e1004231. doi: 10.1371/journal.pgen.1004231 (PMC3961195; doi:10.1371/journal.pgen.1004231)

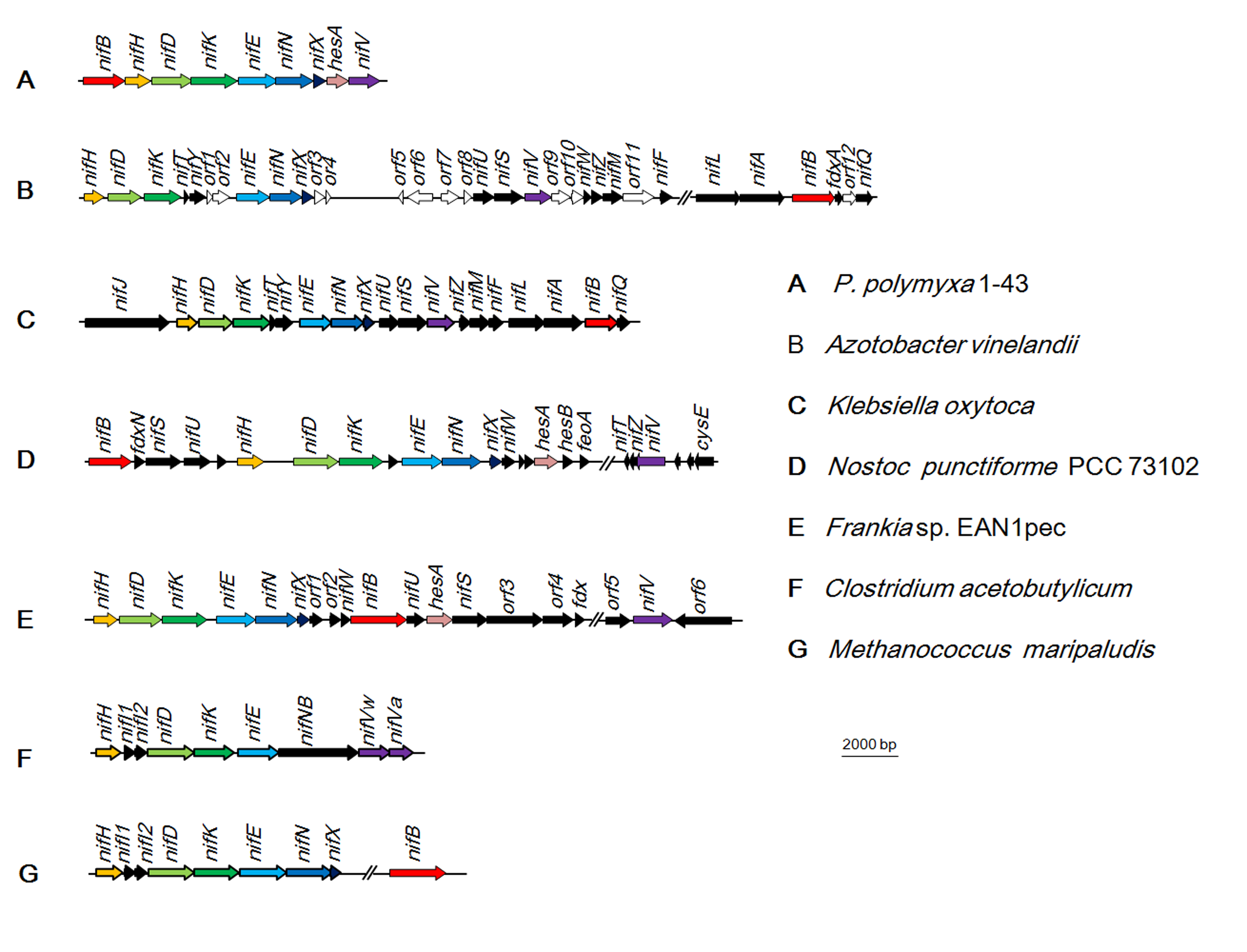

Supplement: Figure S1 — Comparison of the nif gene cluster of Paenibacillus with those of the representative N2-fixing bacteria and archaea. (A) Paenibacillus polymyxa 1–43, (B) Azotobacter vinelandii, (C) Klebsiella oxytoca M5al, (D) Nostoc punctiforme PCC 73102, (E) Frankia sp. EAN1pec, (F) Clostridium acetobutylicum, (G) Methanococus maripaludis. (TIF) [file pgen.1004231.s001.tif]

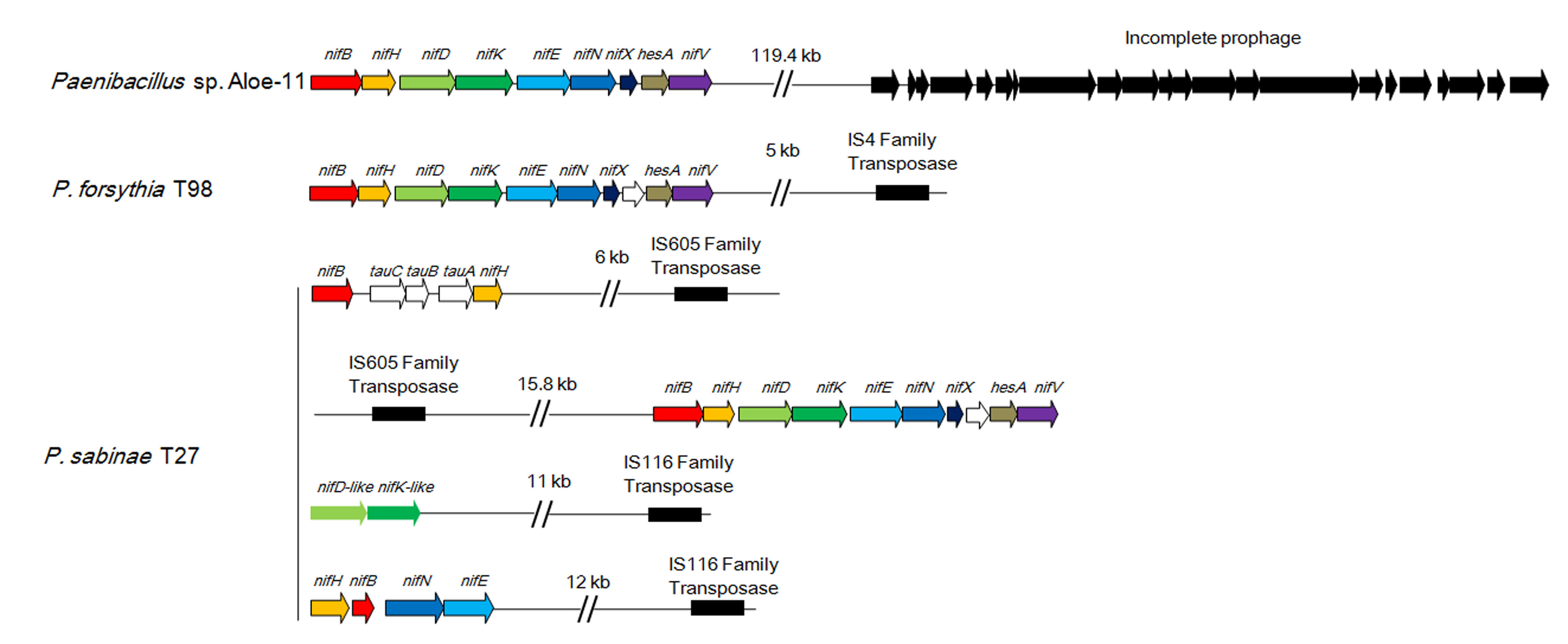

Supplement: Figure S2 — IS elements or prophages linked with the nif gene, nif cluster and nif-like genes. (TIF) [file pgen.1004231.s002.tif]

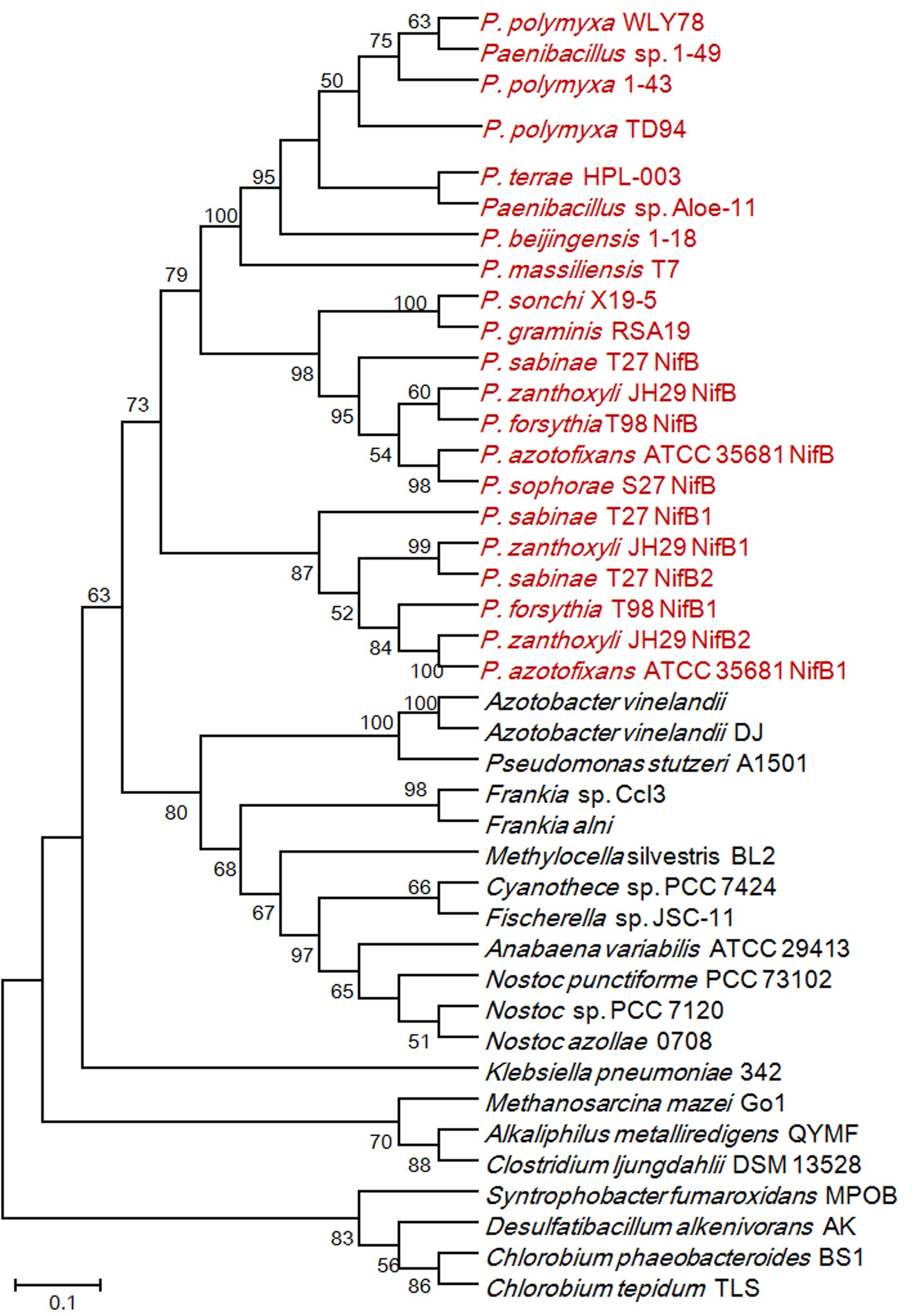

Supplement: Figure S3 — Neighbor joining phylogenetic tree of the NifB sequences derived from Paenibacillus and other representative species. A total of 1,000 bootstrap replicates were made, and bootstrap values are indicated at each node. (TIF) [file pgen.1004231.s003.tif]

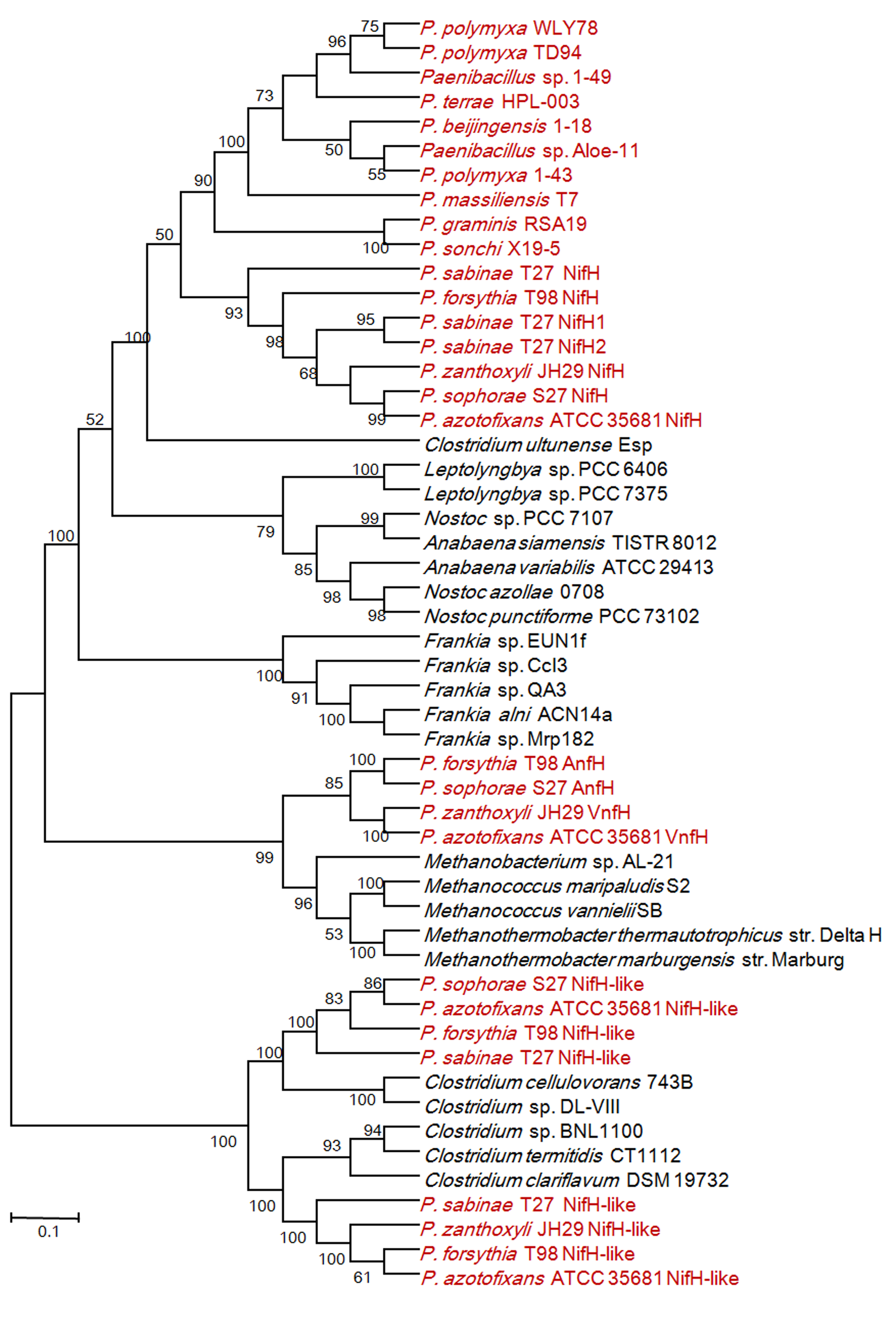

Supplement: Figure S4 — Neighbor joining phylogenetic tree of the NifH, VnfH, AnfH and NifH-like protein sequences derived from Paenibacillus and other representative species. A total of 1,000 bootstrap replicates were made, and bootstrap values are indicated at each node. (TIF) [file pgen.1004231.s004.tif]

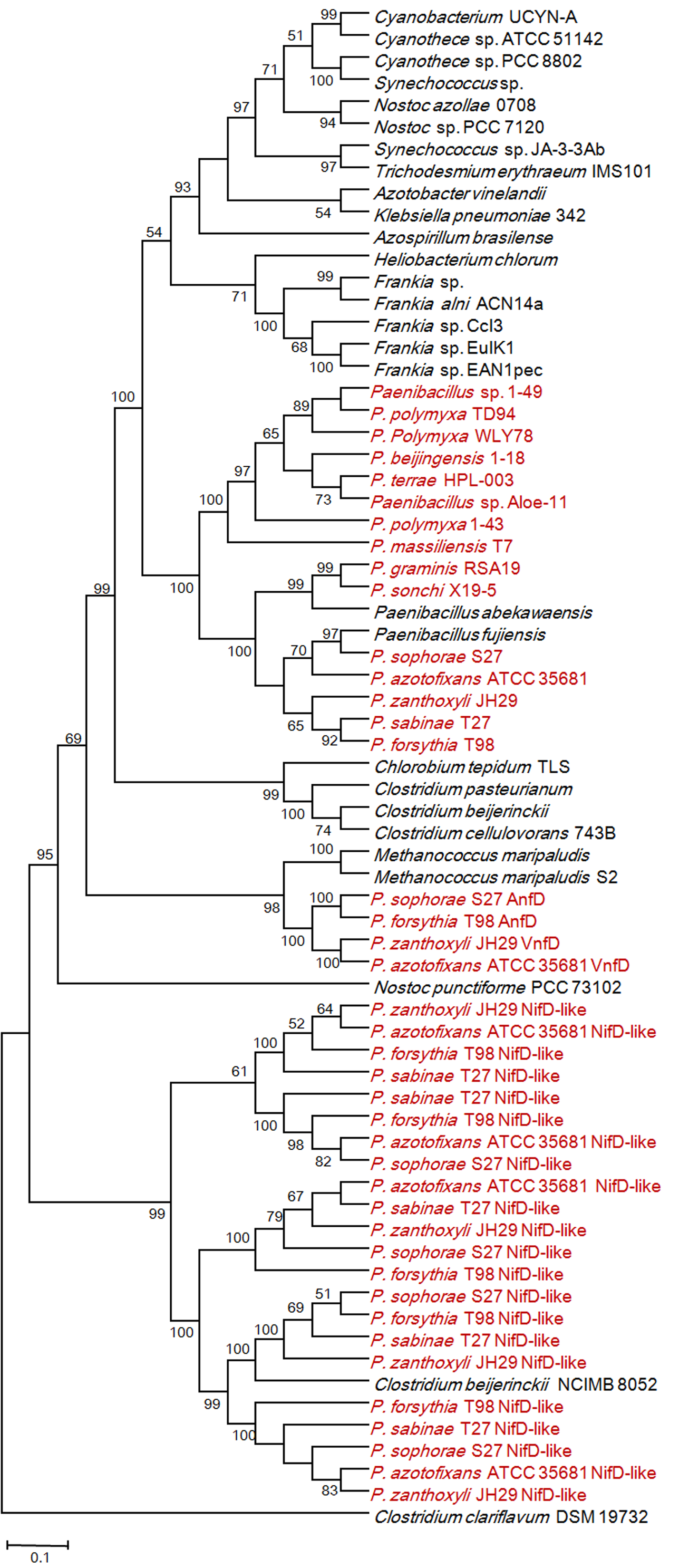

Supplement: Figure S5 — Neighbor joining phylogenetic tree of the NifD, VnfD, AnfD and NifD-like protein sequences derived from Paenibacillus and other representative species. A total of 1,000 bootstrap replicates were made, and bootstrap values are indicated at each node. (TIF) [file pgen.1004231.s005.tif]

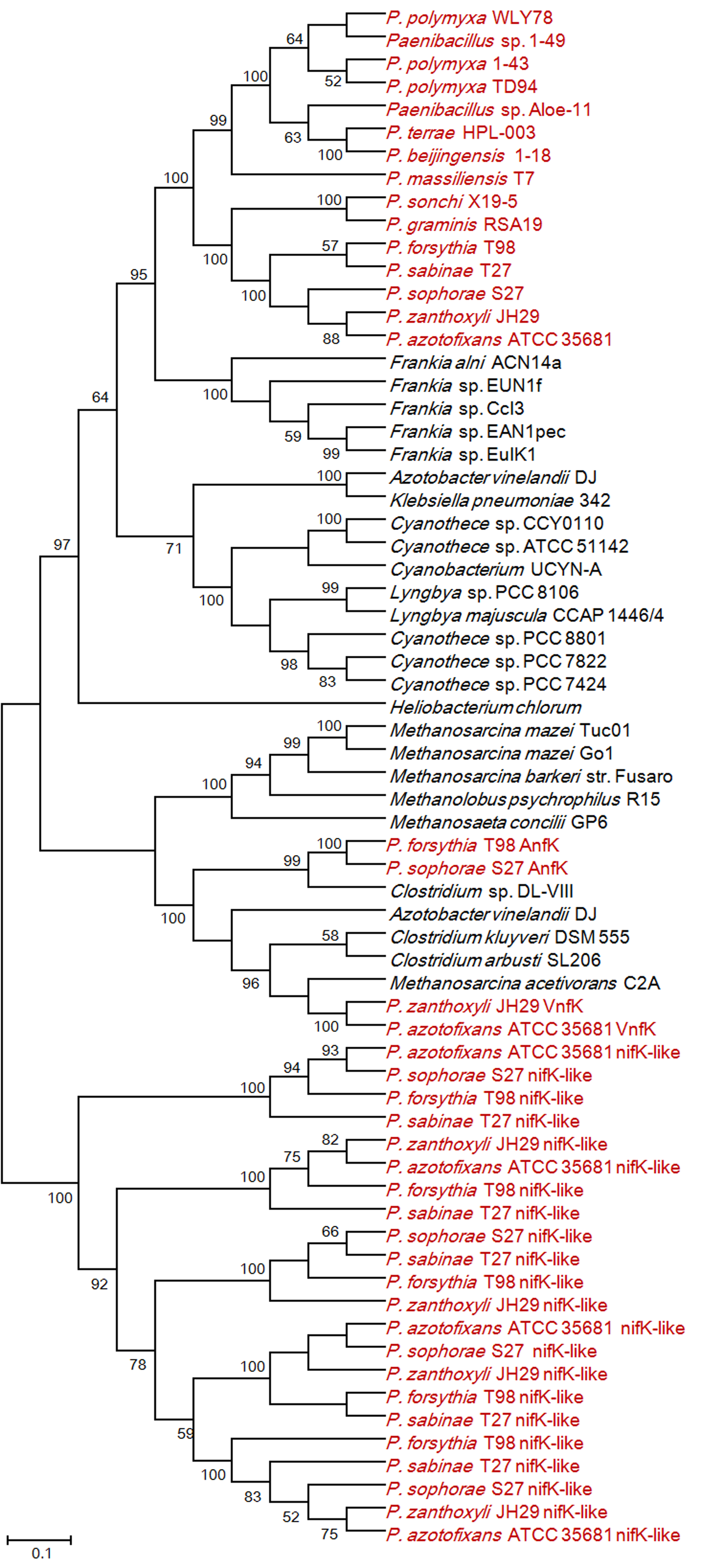

Supplement: Figure S6 — Neighbor joining phylogenetic tree of the NifK, VnfK, AnfK and NifK-like protein sequences derived from Paenibacillus and other representative species. A total of 1,000 bootstrap replicates were made, and bootstrap values are indicated at each node. (TIF) [file pgen.1004231.s006.tif]

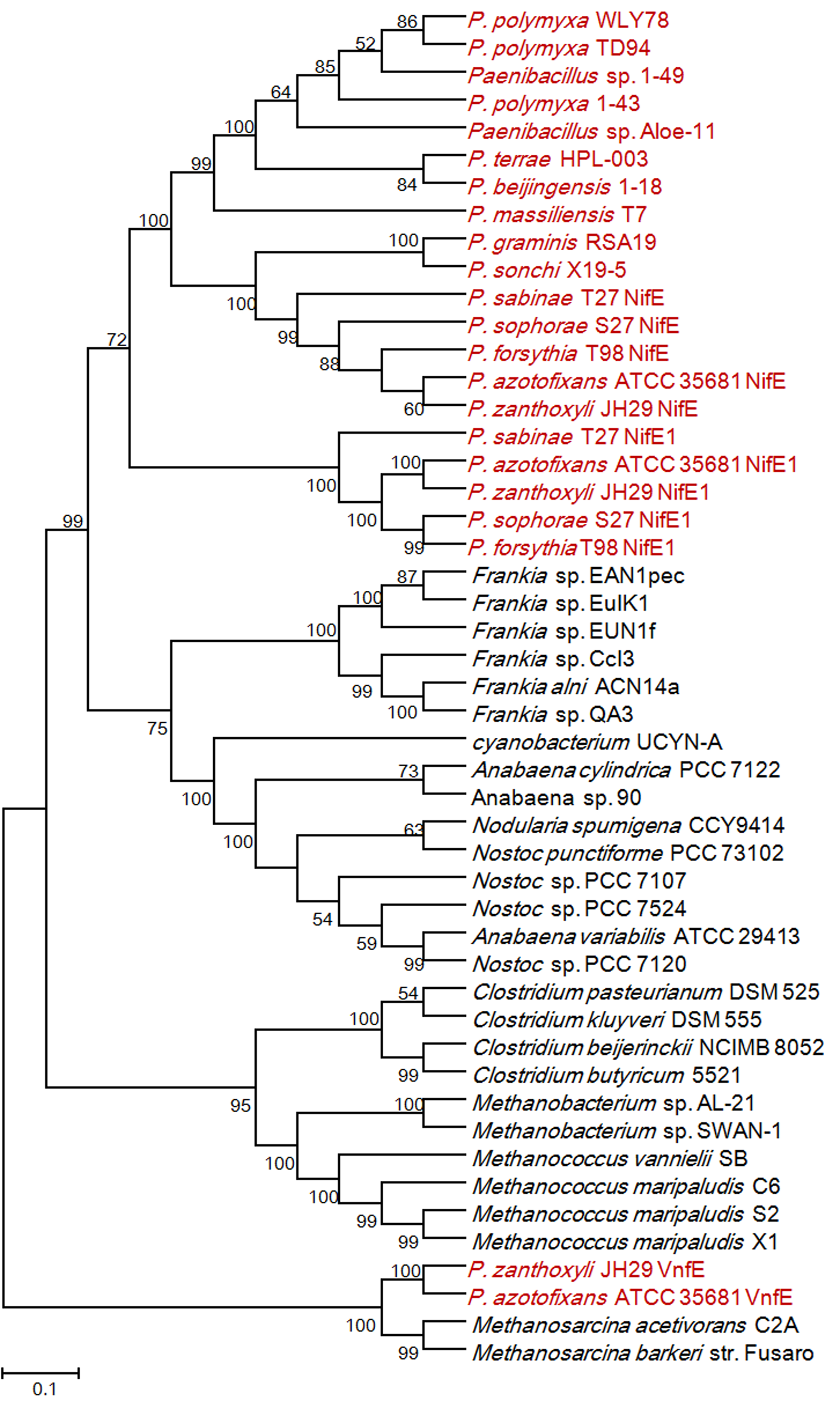

Supplement: Figure S7 — Neighbor joining phylogenetic tree of the NifE, VnfE and NifE-like protein sequences derived from Paenibacillus and other representative species. A total of 1,000 bootstrap replicates were made, and bootstrap values are indicated at each node. (TIF) [file pgen.1004231.s007.tif]

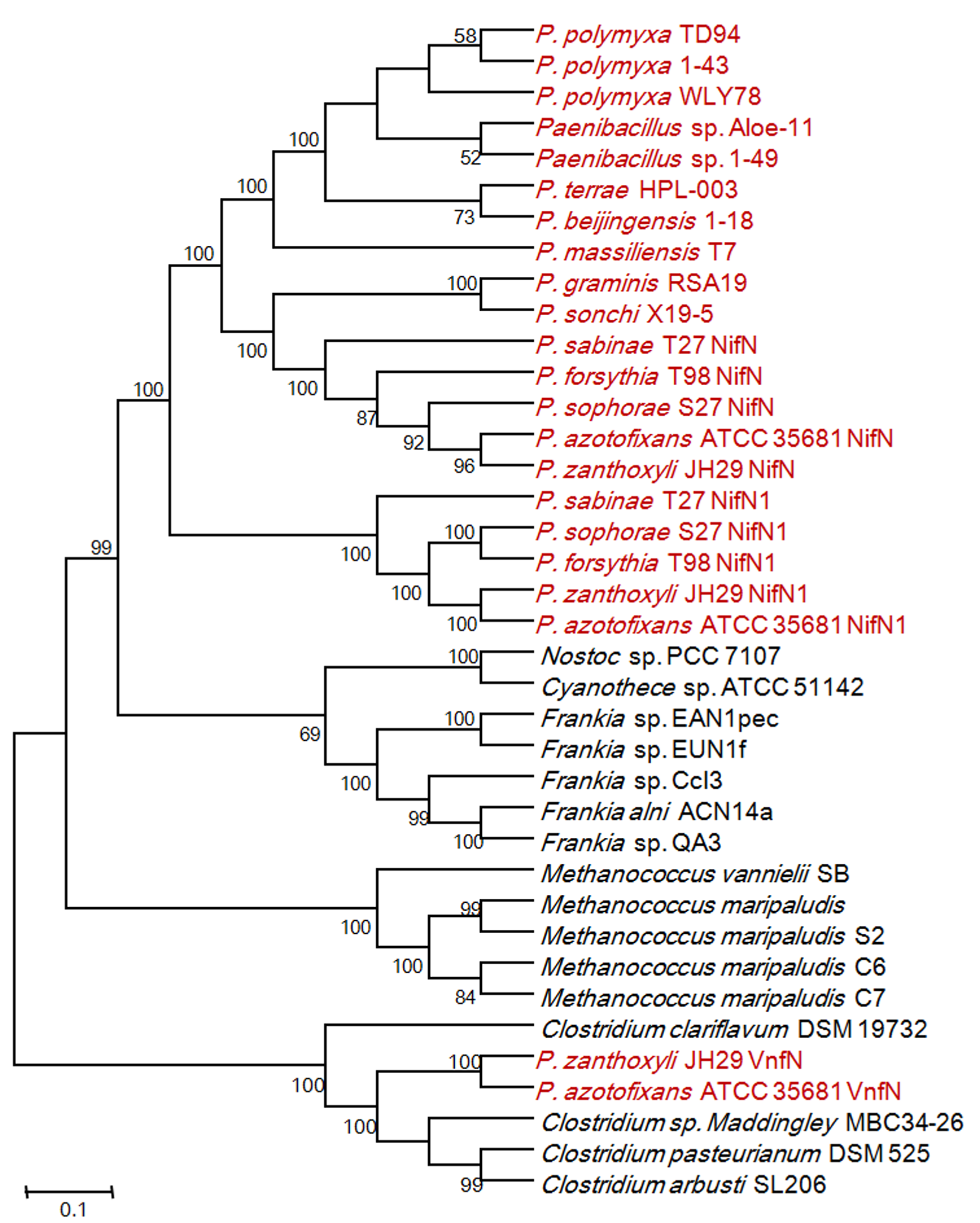

Supplement: Figure S8 — Neighbor joining phylogenetic tree of the NifN, VnfN and NifN-like protein sequences derived from Paenibacillus and other representative species. A total of 1,000 bootstrap replicates were made, and bootstrap values are indicated at each node. (TIF) [file pgen.1004231.s008.tif]

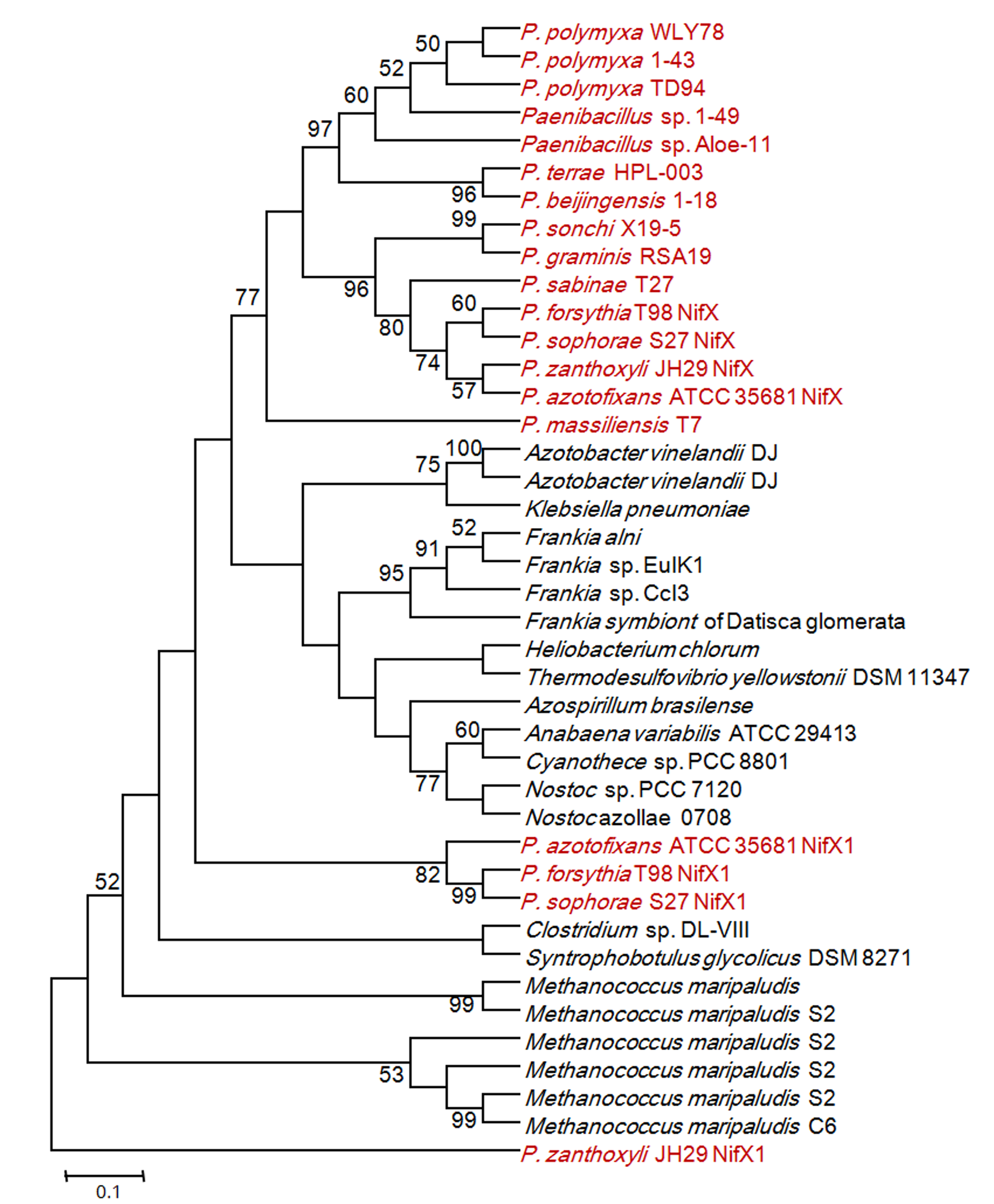

Supplement: Figure S9 — Neighbor joining phylogenetic tree of the NifX protein sequences derived from Paenibacillus and other representative species. A total of 1,000 bootstrap replicates were made, and bootstrap values are indicated at each node. (TIF) [file pgen.1004231.s009.tif]

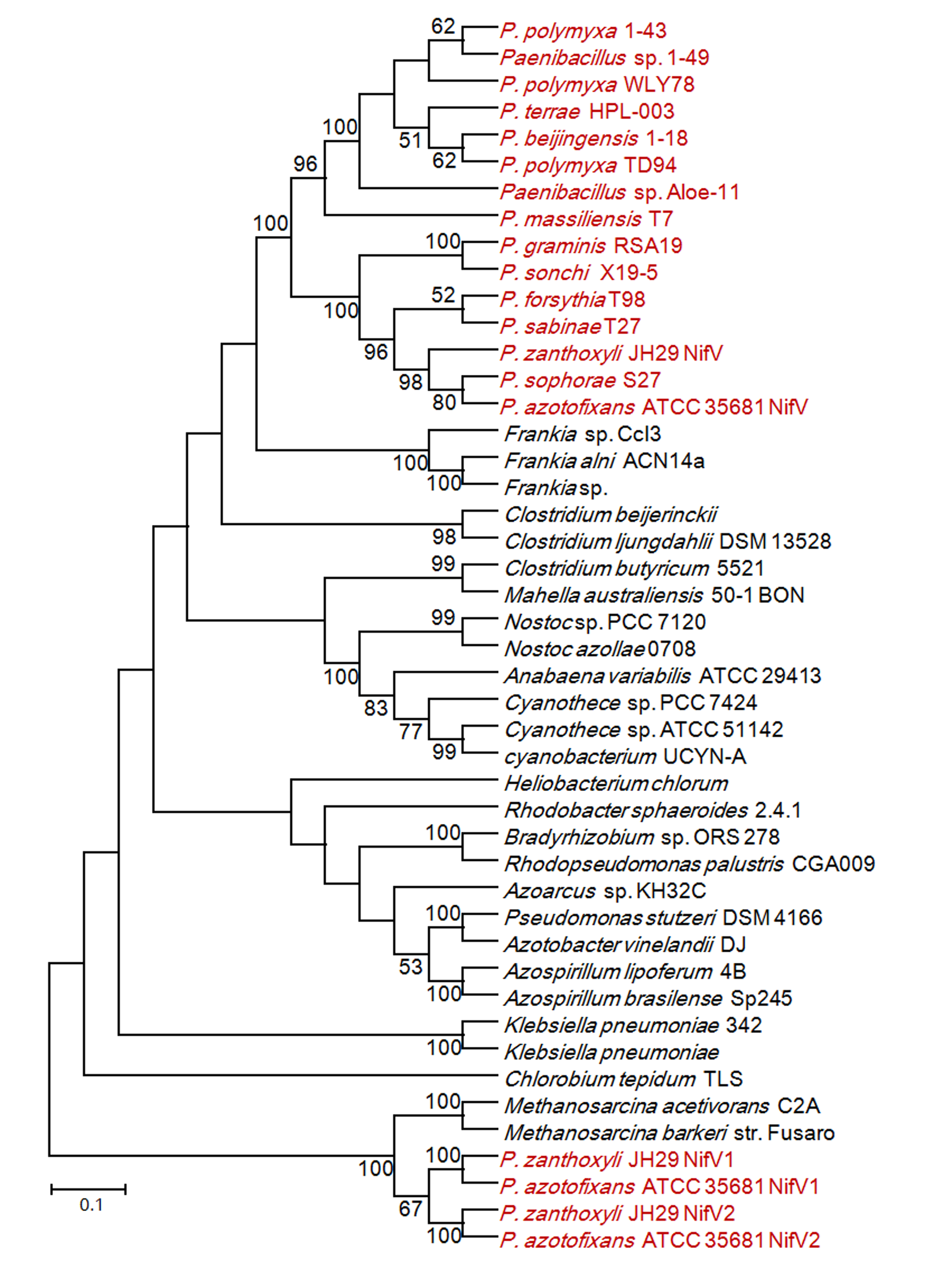

Supplement: Figure S10 — Neighbor joining phylogenetic tree of the NifV protein sequences derived from Paenibacillus and other representative species. A total of 1,000 bootstrap replicates were made, and bootstrap values are indicated at each node. (TIF) [file pgen.1004231.s010.tif]

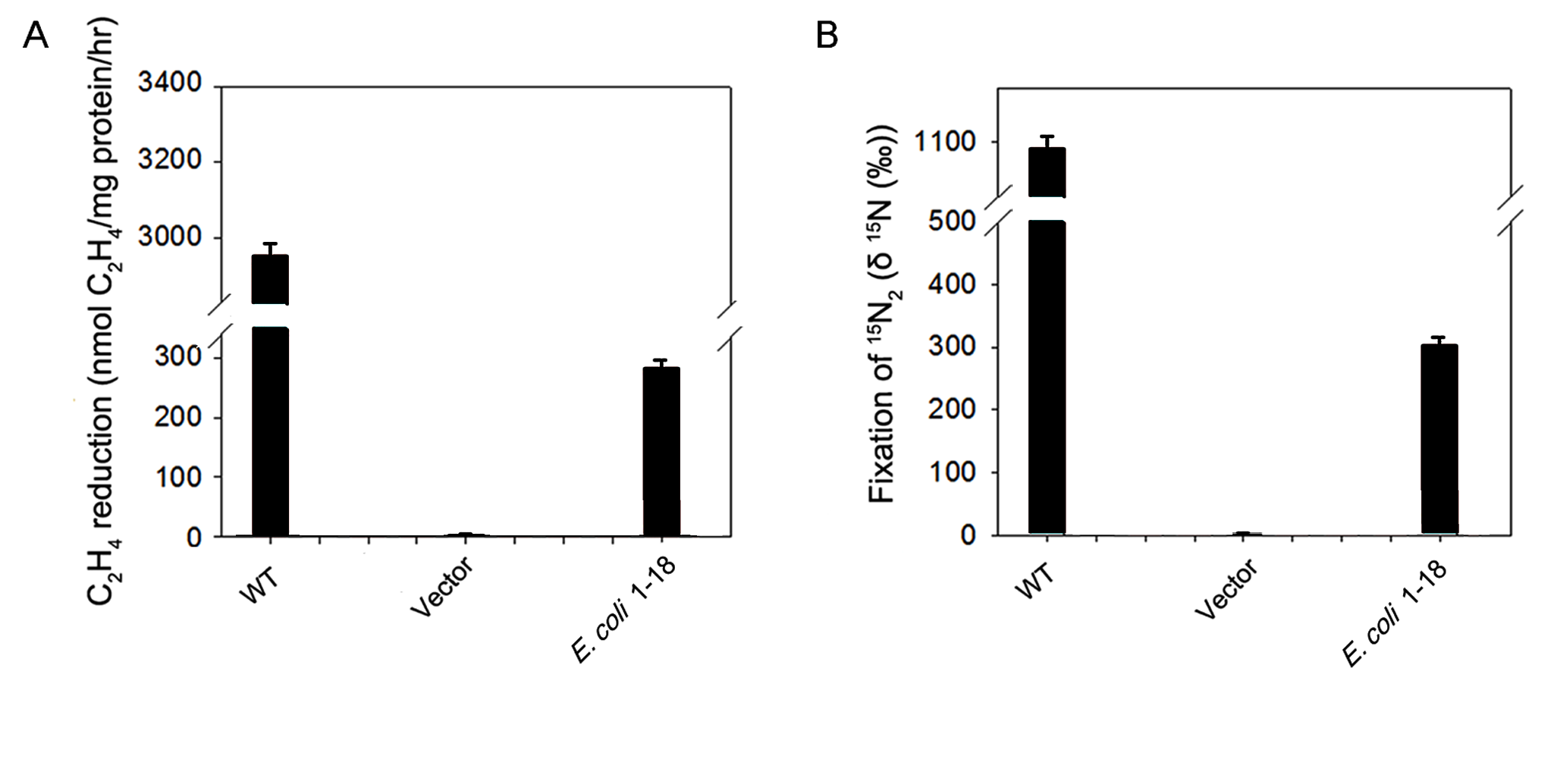

Supplement: Figure S11 — Nitrogen fixation abilities of P. beijingensis 1–18 (WT) and recombinant E. coli 1–18 strain. (A) Nitrogenase activities determined by using acetylene reduction assay. (B) Nitrogen fixation ability determined by using for 15N2 incorporation. Error bars indicate the standard deviation observed from at least two independent experiments. (TIF) [file pgen.1004231.s011.tif]

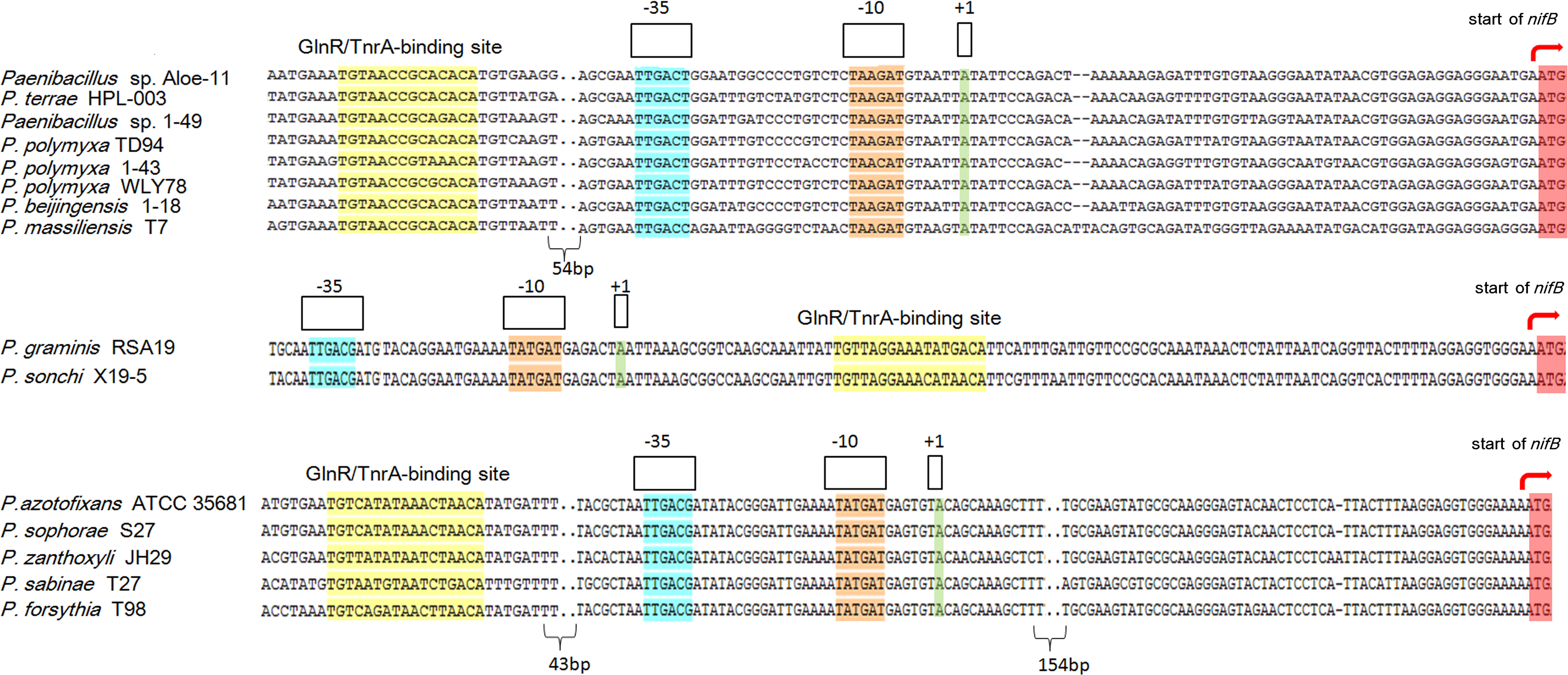

Supplement: Figure S12 — The σ70-depedent promoters of the nif clusters and the GlnR/TnrA-binding sites in the nif promoter regions in Paenibacillus strains. (TIF) [file pgen.1004231.s012.tif]

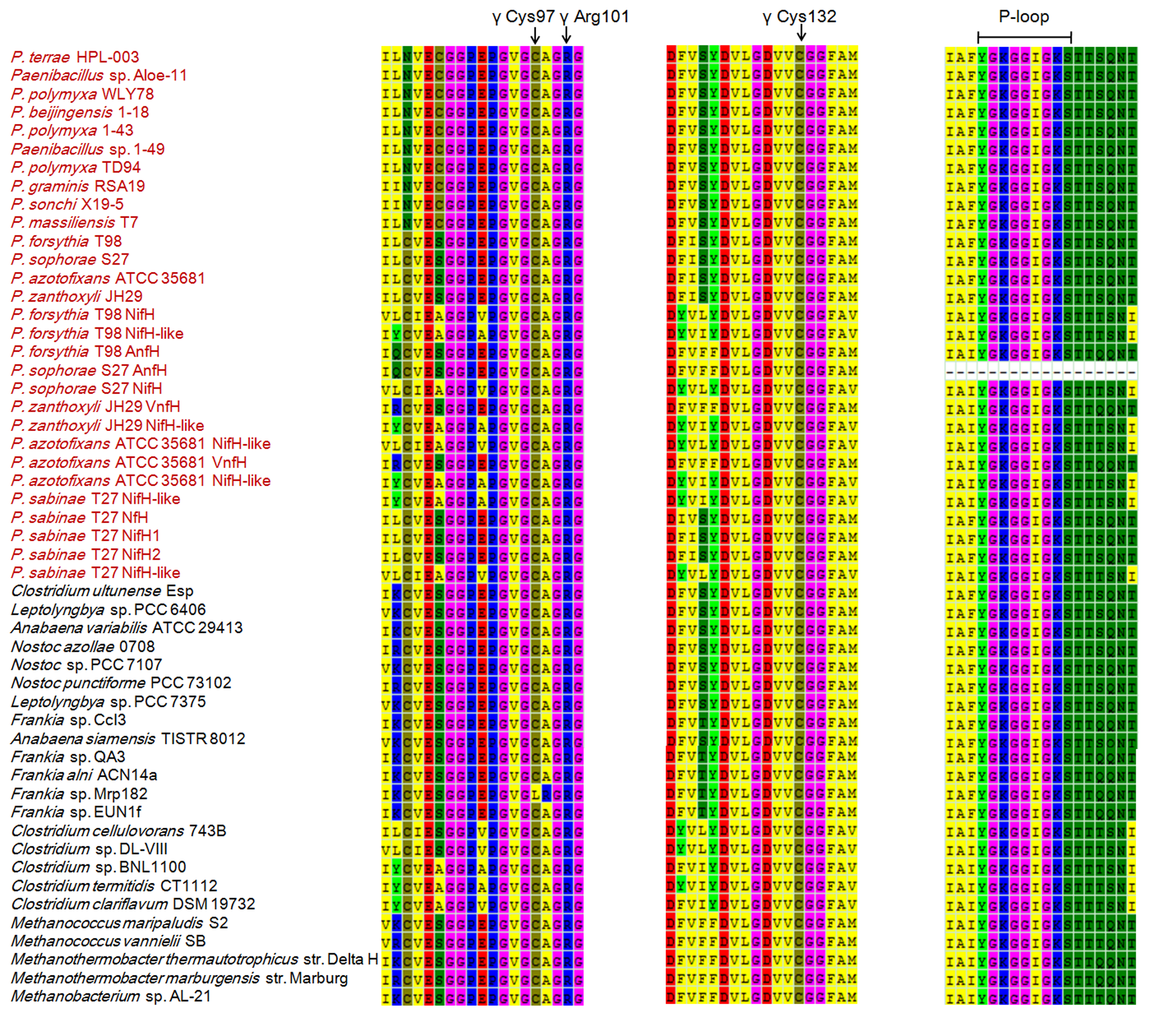

Supplement: Figure S13 — Alignments of crucial residues surrounding the P-loop/MgATP binding motif, cysteine ligating 4Fe-4S and arginine ligating ADP-ribose in NifH and NifH-like protein sequences from Paenibacillus and other organisms. (TIF) [file pgen.1004231.s013.tif]

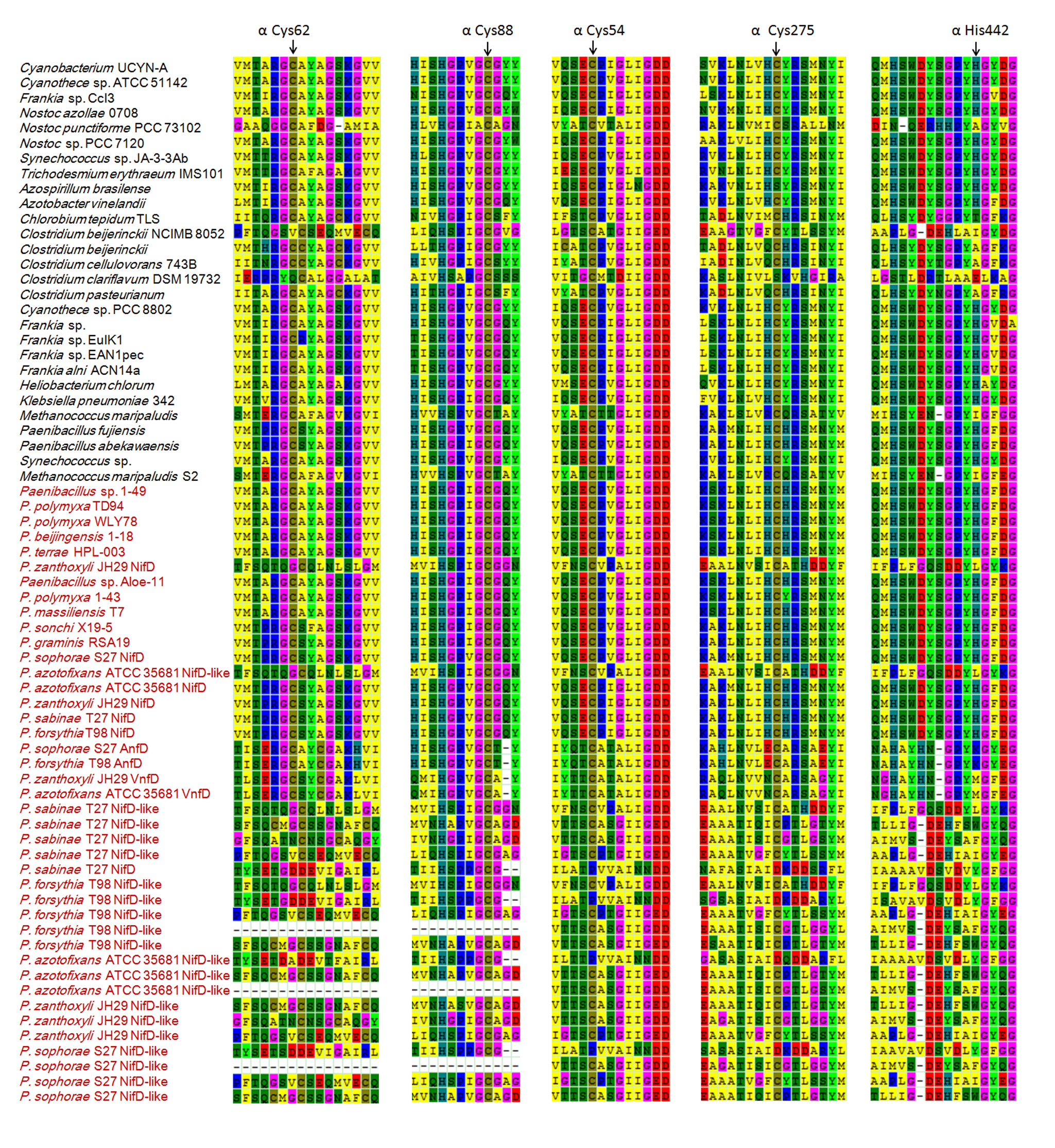

Supplement: Figure S14 — Alignments of crucial residues ligating FeMo-co or P-cluster in NifD and NifD-like protein sequences from Paenibacillus and other organisms. (TIF) [file pgen.1004231.s014.tif]

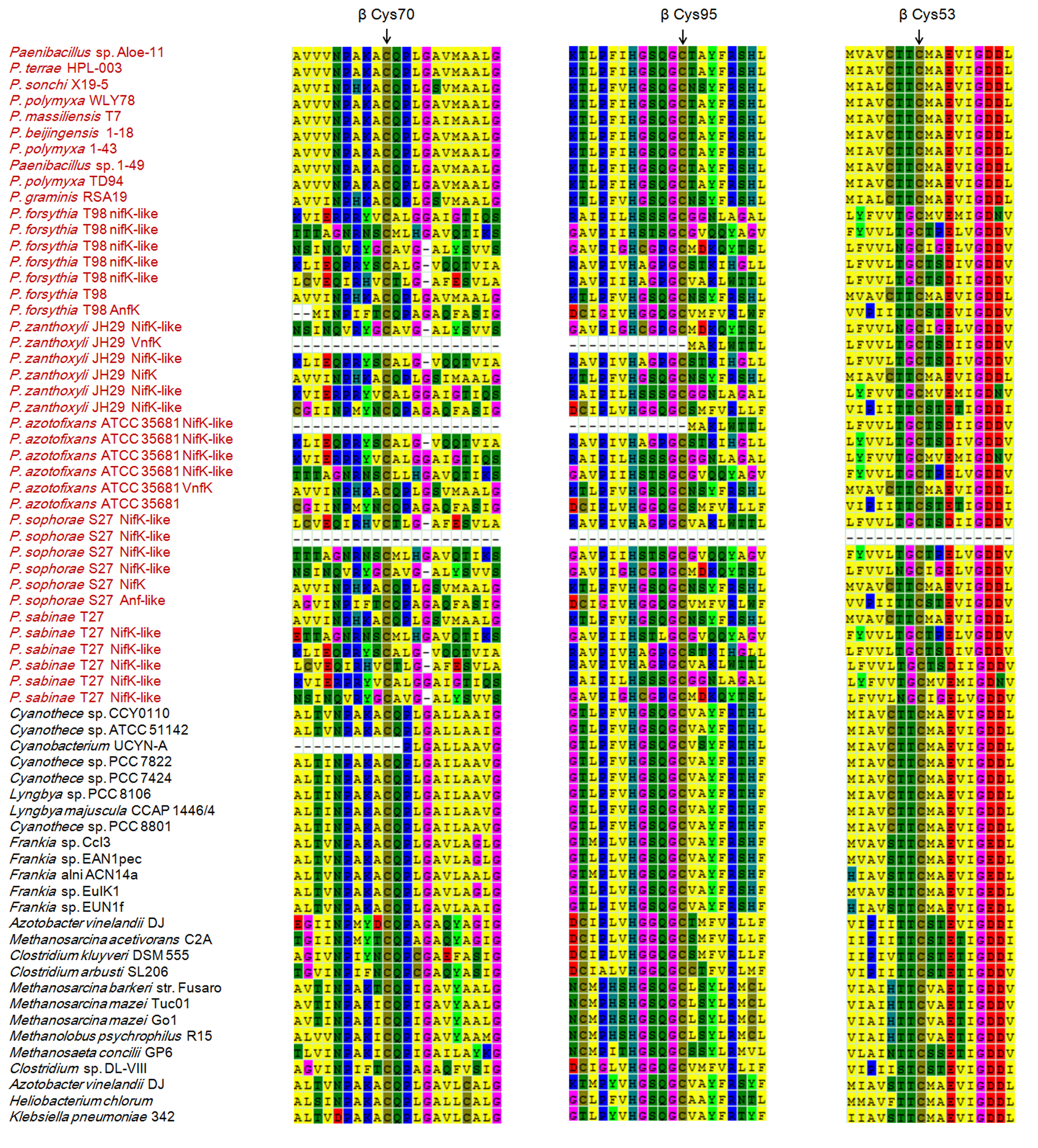

Supplement: Figure S15 — Alignments of crucial residues ligating FeMo-co or P-cluster in in NifK and NifK-like protein sequences from Paenibacillus and other organisms. (TIF) [file pgen.1004231.s015.tif]
