# Supplementary material for: Comparative Genomic Analysis of N2-Fixing and Non-N2-Fixing Paenibacillus spp.: Organization, Evolution and Expression of the Nitrogen Fixation Genes
Source: PLoS Genet. 2014 Mar 20;10(3):e1004231. doi: 10.1371/journal.pgen.1004231 (PMC3961195; doi:10.1371/journal.pgen.1004231)
Supplement: Table S1 — The genomes sequenced in this study. (DOCX) [file pgen.1004231.s016.docx]

| Strains |  | Contigs | | |  | | Scaffolds | | | | |  | | Clean Data | | | |  | Error ratio | |
| --- | --- | --- | --- | --- | --- | --- | --- | --- | --- | --- | --- | --- | --- | --- | --- | --- | --- | --- | --- | --- |
|  |  | No. | Size (Mb) | N50 (kb) |  | | No. | Size (Mb) | | N50 (kb) | |  | | Depth | | | |  |  |  |
| *P.azotofixans* ATCC 35681 |  | 974 | 5.39 | 13.17 |  | 386 | | | 5.44 | | 50.96 | |  | | 110.3 | |  | | | 2.13 |
| *P. graminis* RSA19 |  | 948 | 7.02 | 16.41 |  | 171 | | | 7.08 | | 105.81 | |  | | 84.8 | |  | | | 0.69 |
| *P. sophorae* S27 |  | 1279 | 8.45 | 15.41 |  | 404 | | | 8.52 | | 72.56 | |  | | 70.4 |  | | | | 1.19 |
| *P. sonchi* X19-5 |  | 1440 | 7.52 | 12.94 |  | 378 | | | 7.61 | | 62.20 | |  | | 78.9 | |  | | | 1.11 |
| *P. polymyxa* WLY78 |  | 303 | 5.91 | 69.08 |  | 87 | | | 5.93 | | 334.03 | |  | | 101.3 | |  | | | 0.82 |
| *P. massiliensis* T7 |  | 328 | 6.30 | 46.22 |  | 95 | | | 6.32 | | 239.44 | |  | | 94.9 | |  | | | 0.48 |
| *P. zanthoxyli* JH29 |  | 866 | 5.08 | 13.81 |  | 307 | | | 5.12 | | 42.88 | |  | | 117.2 | |  | | | 2.7 |
| *P. forsythia* T98 |  | 1136 | 5.13 | 11.07 |  | 295 | | | 5.19 | | 52.37 | |  | | 115.5 | |  | | | 1.74 |
| *P. beijingensis* 1-18 |  | 489 | 5.42 | 31.25 |  | 203 | | | 5.44 | | 106.98 | |  | | 110.3 | |  | | | 1.45 |
| *P. polymyxa* TD94 |  | 822 | 6.06 | 34.13 |  | 462 | | | 6.10 | | 170.03 | |  | | 98.4 | |  | | | 4.95 |
| *P. polymyxa* 1-43 |  | 297 | 5.98 | 62.02 |  | 98 | | | 6.00 | | 220.66 | |  | | 100.0 | |  | | | 0.72 |
| *Paenibacillus* sp. 1-49 |  | 413 | 5.63 | 42.69 |  | 143 | | | 5.65 | | 155.43 | |  | | 106.2 | |  | | | 0.72 |
