# Supplementary material for: Comparative Genomic Analysis of N2-Fixing and Non-N2-Fixing Paenibacillus spp.: Organization, Evolution and Expression of the Nitrogen Fixation Genes
Source: PLoS Genet. 2014 Mar 20;10(3):e1004231. doi: 10.1371/journal.pgen.1004231 (PMC3961195; doi:10.1371/journal.pgen.1004231)
Supplement: Table S2 — Comparison of COG assignments between non-N2-fixing and N2-fixing Paenibacillus strains. (DOCX) [file pgen.1004231.s017.docx]

| Individual functional categories | Core genes | | | Specific genes † | |
| --- | --- | --- | --- | --- | --- |
|  | Non | Nif | P-value*** | Non | Nif |
| J: Translation, ribosomal structure and biogenesis | 96 | 91 | 0.01349 | 0 | 0 |
| K: Transcription | 67 | 76 | 0.2559 | 0 | 0 |
| L: DNA Replication, recombination, and repair | 63 | 58 | 0.02954 | 0 | 0 |
| D: Cell division and chromosome partitioning | 12 | 20 | 0.7194 | 0 | 0 |
| V: Denfense mechanisms | 7 | 11 | 1 | 0 | 0 |
| T: Signal transduction mechanisms | 28 | 49 | 0.4104 | 0 | 0 |
| M: Cell envelope biogenesis, outer membrane | 43 | 71 | 0.4358 | 0 | 0 |
| N: Cell motility and chemotaxis | 4 | 33 | 6.71E-05 | 0 | 0 |
| U: Intracellular trafficking, secretion, and vesicular transport | 14 | 25 | 0.5148 | 0 | 0 |
| O: Posttranslational modification, protein turnover, chaperones | 41 | 50 | 0.588 | 0 | 0 |
| C: Energy production and conversion | 39 | 58 | 0.8335 | 0 | 4 |
| E: Amino acid transport and metabolism | 80 | 104 | 0.6974 | 0 | 1 |
| F: Nucleotide transport and metabolism | 39 | 50 | 0.7427 | 0 | 0 |
| G: Carbohydrate transport and metabolism | 42 | 65 | 0.6173 | 0 | 0 |
| H: Coenzyme transport and metabolism | 60 | 67 | 0.2308 | 0 | 1 |
| I: Lipid metabolism | 25 | 23 | 0.1831 | 0 | 0 |
| Q: Secondary metabolites biosynthesis, transport and catabolism | 7 | 11 | 1 | 0 | 0 |
| P: Inorganic ion transport and metabolism | 42 | 52 | 0.5947 | 0 | 1 |
| R: General function prediction only | 96 | 146 | 0.5368 | 0 | 2 |
| S: Function unknown | 76 | 115 | 0.6461 | 0 | 0 |
