# Supplementary material for: Comparative Genomic Analysis of N2-Fixing and Non-N2-Fixing Paenibacillus spp.: Organization, Evolution and Expression of the Nitrogen Fixation Genes
Source: PLoS Genet. 2014 Mar 20;10(3):e1004231. doi: 10.1371/journal.pgen.1004231 (PMC3961195; doi:10.1371/journal.pgen.1004231)
Supplement: Table S3 — Transposons present in the genomes of 31 Paenibacillus strains. The following information is provided for each putative transposon in genomes: transposon family, transposases, and numbers of copies of intact or remnant transposons in each genome. (DOCX) [file pgen.1004231.s018.docx]

| Type | Family | Copies |
| --- | --- | --- |
| ***Paenibacillus* sp. JDR2** |  |  |
| ISBspe1 | IS1182 | 5 |
| ISFnu1 | IS1182 | 3 |
| ***Paenibacillus* sp. Y412MC10** |  |  |
| ISVsa5 | IS4 | 2 |
| IS10R | IS4 | 1 |
| ISBsu1 | IS3 | 4 |
| ISErh1 | IS3 | 2 |
| ISSsu4 | IS200/IS605 | 2 |
| ***P. mucilaginosus* KNP414** |  |  |
| ISBcy1 | IS1182 | 7 |
| ISBsu1 | IS3 | 1 |
| ISBse2 | IS1182 | 6 |
| ISErh1 | IS3 | 1 |
| ***P. mucilaginosus* K02** |  |  |
| ISBcy1 | IS1182 | 8 |
| ISBse2 | IS1182 | 7 |
| ISBsu1 | IS3 | 1 |
| ISErh1 | IS3 | 1 |
| ***P. mucilaginosus* 3016** |  |  |
| ISBcy1 | IS1182 | 8 |
| ISBsu1 | IS3 | 1 |
| ISBse2 | IS1182 | 7 |
| ISErh1 | IS3 | 1 |
| ISCce4 | IS3 | 1 |
| ***P. polymyxa* SC2** |  |  |
| ISBsu1 | IS3/IS911 | 1 |
| ISBli1 | ISl3 | 1 |
| ISVsa5 | IS4 | 1 |
| ***P. polymyxa* E681** |  |  |
| ISBsu1 | IS3 | 3 |
| ISErh1 | IS3 | 1 |
| IS655 | IS3 | 1 |
| ISSmi2 | IS1182 | 2 |
| ISBspe1 | IS1182 | 1 |
| ISClsp1 | IS3 | 1 |
| ISHaha5 | IS110 | 1 |
| IS658 | IS30 | 1 |
| ***P. terrae* HPL-003** |  |  |
| ISBth16 | IS200/IS605 | 2 |
| ISClsp1 | IS3 | 4 |
| ISBce18 | IS3 | 1 |
| ISEfa8 | IS3 | 1 |
| ***P. curdlanolyticus* YK9** |  |  |
| ISBspe1 | IS1182 | 5 |
| ISBbr1 | IS1182 | 2 |
| ISBspe1 | IS1182 | 1 |
| ISBth8 | IS3 | 2 |
| ***Paenibacillus* sp. Aloe-11** |  |  |
| ISHaha5 | IS110 | 2 |
| ISBth10 | IS3 | 1 |
| ISCod1 | IS3 | 1 |
| ISFsp8 | IS3 | 1 |
| ***Paenibacillus* sp. HGF5** |  |  |
| ISPsy8 | IS3 | 2 |
| ISBce18 | IS3 | 3 |
| ISBth10 | IS3 | 3 |
| ISBce19 | IS3 | 2 |
| ISBth167 | IS3 | 1 |
| ISRme12 | IS3 | 1 |
| ISBam1 | IS3 | 1 |
| ***Paenibacillus* sp. HGF7** |  |  |
| ISBse1 | IS1595 | 4 |
| ISBth10 | IS3 | 1 |
| ISBce18 | IS3 | 1 |
| ISBth167 | IS3 | 1 |
| ISBce19 | IS3 | 1 |
| ISMno23 | IS91 | 1 |
| ISBsp3 | IS3 | 1 |
| ***P. dendritiformis* C454** |  |  |
| ISBsp2 | IS3 | 3 |
| ISBce14 | IS3 | 1 |
| ISCpe6 | IS3 | 1 |
| ISFjo2 | IS3 | 1 |
| ISClte1 | IS3 | 1 |
| ISCb4 | IS3 | 1 |
| ISBsu1 | IS3 | 1 |
| ISErh1 | IS3 | 1 |
| ISTte1 | IS1182 | 1 |
| ISDha13 | IS200/IS605 | 1 |
| IS1182 | IS1182 | 1 |
| ISBce1 | IS1182 | 1 |
| ISBthe2 | IS1182 | 1 |
| IS660 | IS1182 | 1 |
| ***P. elgii* B69** |  |  |
| ISBcy1 | IS1182 | 2 |
| ISBse2 | IS1182 | 1 |
| ISBsp4 | IS1182 | 1 |
| ISDha11 | IS1182 | 2 |
| ISDha8 | IS1182 | 1 |
| ISPa40 | Tn3 | 1 |
| ISBsu1 | IS3 | 2 |
| ISErh1 | IS3 | 1 |
| ISBbr1 | IS1182 | 5 |
| ISSac1 | IS630 | 1 |
| ISAcsp1 | Tn3 | 1 |
| ISArsp6 | Tn3 | 1 |
| *P. lactis*154 |  |  |
| ISPeth1 | ISNCY | 3 |
| IS200S | IS200/IS605 | 1 |
| ISShes11 | Tn3 | 1 |
| ***P. peoriae* KCTC 3763** |  | 1 |
| ISLtaq1 | IS3 | 2 |
| ISBth8 | IS3 | 1 |
| ISBce18 | IS3 | 2 |
| ISBth10 | IS3 | 1 |
| ISCth10 | IS200/IS605 | 1 |
| ISDha13 | IS200/IS605 | 1 |
| ISSsu4 | IS200/IS605 | 1 |
| IS655 | IS3 | 1 |
| ***Paenibacillus* sp. oral taxon786D14** |  |  |
| ISBsu1 | IS3 | 2 |
| ISGka1 | IS5 | 1 |
| ISBbr1 | IS1182 | 4 |
| ISBs2 | IS630 | 1 |
| ISPaen1 | IS1380 | 1 |
| ISPaen2 | IS5 | 1 |
| ISBco3 | IS5 | 1 |
| ISBth17 | IS200/IS605 | 4 |
| ISHaha7 | IS200/IS605 | 1 |
| ISPaen3 | IS5 | 1 |
| ISVha3 | IS5 | 1 |
| ISSpn10 | IS110 | 1 |
| ISGka1 | IS5 | 2 |
| IS655 | IS3 | 1 |
| ISClsp1 | IS3 | 1 |
| ISEfa8 | IS3 | 1 |
| ISBsu1 | IS3 | 1 |
| ISSpn10 | IS110 | 2 |
| ISBth166 | IS110 | 1 |
| ISSpn10 | IS110 | 1 |
| ***P. vortex* V453** |  |  |
| ISShes11 | Tn3 | 2 |
| ISBsu1 | IS3 | 2 |
| ISErh1 | IS3 | 1 |
| ISBsu1 | IS3 | 1 |
| ISBce18 | IS3 | 7 |
| ISBsu1 | IS3 | 2 |
| ISErh1 | IS3 | 1 |
| ISRme12 | IS3 | 1 |
| ISBope1 | IS3 | 1 |
| ISBth10 | IS3 | 1 |
| ISPaen1 | IS1380 | 1 |
| ISSsu4 | IS200/IS605 | 1 |
| IS200S | IS200/IS605 | 1 |
| ISBce19 | IS3 | 6 |
| ISBth10 | IS3 | 3 |
| ISBth167 | IS3 | 1 |
| ISHaha3 | IS3 | 1 |
| ISBce18 | IS3 | 1 |
| ***P. azotofixans* ATCC 35681** |  |  |
| IS231U | IS4 | 2 |
| IS231F2 | IS4 | 1 |
| IS231F | IS4 | 1 |
| ISDre4 | IS1380 | 1 |
| ISEfa4 | IS200/IS605 | 1 |
| ISMsp1 | IS1182 | 1 |
| ISBth15 | IS200/IS605 | 2 |
| ISDha13 | IS200/IS605 | 1 |
| ***P. graminis* RSA19** |  |  |
| ISBce1 | IS1182 | 2 |
| ISSmi2 | IS1182 | 1 |
| ISBf5 | IS1182 | 1 |
| ISHaha5 | IS110 | 1 |
| ISShes11 | Tn3 | 1 |
| ISSsu4 | IS200/IS605 | 1 |
| ISBce3 | IS200/IS605 | 3 |
| ISClte2 | IS200/IS605 | 1 |
| ***P. sophorae* S27** |  |  |
| ISDha13 | IS200/IS605 | 2 |
| ISBse2 | IS1182 | 2 |
| ISBcy1 | IS1182 | 2 |
| ISBsu1 | IS3 | 1 |
| ISBsp4 | IS1182 | 1 |
| ISBas1 | IS1182 | 1 |
| ISMsp1 | IS1182 | 1 |
| ISDha5 | IS4 | 1 |
| ISPssp1 | IS3 | 3 |
| IS1301 | IS5 | 3 |
| ISPar1 | IS3 | 4 |
| ISAma2 | IS3 | 1 |
| ISSod5 | IS256 | 1 |
| ISSba12 | IS256 | 1 |
| ISAcsp1 | Tn3 | 1 |
| ISShes11 | Tn3 | 1 |
| ISPa38 | Tn3 | 1 |
| ISPa40 | Tn3 | 1 |
| ISOih1 | IS1182 | 2 |
| ISBdo1 | IS1182 | 1 |
| ISBsp4 | IS1182 | 1 |
| ISBas1 | IS1182 | 1 |
| ISStma16 | IS5 | 1 |
| IS1480a | IS5 | 1 |
| ISNieu4 | IS5 | 1 |
| IS1480b | IS5 | 1 |
| ISCaa8 | IS5 | 1 |
| ISAzo36 | IS5 | 1 |
| ISNieu4 | IS5 | 1 |
| ISXo3 | IS5 | 1 |
| ISPa14 | IS1 | 1 |
| ISAba3 | IS1 | 1 |
| ISGlo1 | IS3 | 1 |
| ISBce3 | IS200/IS605 | 1 |
| IS1236 | IS3 | 1 |
| ISCARN64 | IS3 | 1 |
| ISAbo1 | IS3 | 1 |
| ISPssp1 | IS3 | 8 |
| ISAma2 | IS3 | 1 |
| ISAtu5 | IS3 | 1 |
| ISRm6 | IS3 | 1 |
| ISSba13 | IS3 | 1 |
| ISSpu6 | IS3 | 1 |
| ISAba15 | IS5 | 14 |
| ISBcy1 | IS1182 | 1 |
| ISSod18 | IS256 | 1 |
| IS285 | IS256 | 1 |
| IS1414 | IS256 | 1 |
| ISEc39 | IS256 | 1 |
| ISAba2 | IS3 | 2 |
| ISAba19 | IS3 | 4 |
| ISAba18 | IS3 | 2 |
| IS1240 | IS3 | 1 |
| ISPssp2 | IS1 | 5 |
| IS1301 | IS5 | 2 |
| ISPa14 | IS1 | 1 |
| ISAba2 | IS3 | 2 |
| IS1599 | IS982 | 1 |
| ISPa14 | IS1 | 2 |
| IS1592 | IS982 | 2 |
| ISAba3 | IS1 | 2 |
| ISSod20 | IS982 | 1 |
| IS1599 | IS982 | 2 |
| ISStma16 | IS5 | 1 |
| ISAcma7 | IS256 | 1 |
| IS1480a | IS5 | 1 |
| ISSba12 | IS256 | 1 |
| ISAzo36 | IS5 | 1 |
| ISNieu4 | IS5 | 1 |
| ISIMb1 | IS110 | 1 |
| ISStma16 | IS5 | 1 |
| IS1480a | IS5 | 1 |
| ***P. sonchi* X19-5** |  |  |
| ISSep3 | IS200/IS605 | 3 |
| ISOih2 | IS200/IS605 | 1 |
| IS657 | IS200/IS605 | 1 |
| ISSep3 | IS200/IS605 | 3 |
| ISOih2 | IS200/IS605 | 1 |
| IS657 | IS200/IS605 | 1 |
| ISSac1 | IS630 | 1 |
| ISBdo1 | IS1182 | 1 |
| ISDha13 | IS200/IS605 | 9 |
| ISPca2 | IS3 | 1 |
| ISSsu4 | IS200/IS605 | 2 |
| ISCth10 | IS200/IS605 | 3 |
| ISPlu5 | IS200/IS605 | 1 |
| IS200C | IS200/IS605 | 1 |
| ISAcma39 | IS200/IS605 | 1 |
| IS200H | IS200/IS605 | 1 |
| IS200F | IS200/IS605 | 1 |
| IS200 | IS200/IS605 | 1 |
| ISPlu5 | IS200/IS605 | 1 |
| ISArsp6 | Tn3 | 1 |
| ISPlu5 | IS200/IS605 | 1 |
| ISSsu4 | IS200/IS605 | 1 |
| IS200C | IS200/IS605 | 1 |
| ISBth17 | IS200/IS605 | 1 |
| ***P. polymyxa* WLY78** |  |  |
| ISBpl1 | IS5 | 2 |
| ISBma2 | IS1182 | 1 |
| ISRle11 | IS1182 | 1 |
| ISGho1 | IS1182 | 1 |
| ISOih1 | IS1182 | 1 |
| ISBfun3 | IS1182 | 1 |
| ISHaha5 | IS110 | 1 |
| ISPaen2 | IS5 | 2 |
| ISBco3 | IS5 | 1 |
| ISArsp6 | Tn3 | 1 |
| IS655 | IS3 | 1 |
| ISClsp1 | IS3 | 2 |
| ISEfa8 | IS3 | 2 |
| IS655 | IS3 | 1 |
| IS1485 | IS3 | 1 |
| ISSwo1 | IS3 | 1 |
| ***P. massiliensis* T7** |  |  |
| ISDha13 | IS200/IS605 | 9 |
| ISPlu5 | IS200/IS605 | 1 |
| ISBbr1 | IS1182 | 1 |
| IS655 | IS3 | 1 |
| ISClsp1 | IS3 | 1 |
| ISBce18 | IS3 | 2 |
| ISBth10 | IS3 | 3 |
| ISBce19 | IS3 | 2 |
| ISBth167 | IS3 | 1 |
| ISPlsp1 | IS3 | 1 |
| IS200S | IS200/IS605 | 1 |
| ISSsu4 | IS200/IS605 | 1 |
| ISArsp6 | Tn3 | 1 |
| ISBce1 | IS1182 | 1 |
| ***P. zanthoxyli* JH29** |  |  |
| IS200S | IS200/IS605 | 2 |
| ISEfa4 | IS200/IS605 | 1 |
| ISNpu13 | Tn3 | 1 |
| ISSac1 | IS630 | 1 |
| ISBth166 | IS110 | 1 |
| ISCce4 | IS3 | 1 |
| ISCce3 | IS3 | 1 |
| ISBcy1 | IS1182 | 5 |
| ISBse2 | IS1182 | 1 |
| ISBsp4 | IS1182 | 5 |
| ISEfa4 | IS200/IS605 | 1 |
| ISBse2 | IS1182 | 3 |
| ISAco1 | IS1182 | 2 |
| ISNpu13 | Tn3 | 1 |
| ISGsp2 | IS1380 | 1 |
| ISCARN34 | IS1380 | 1 |
| ISEfa4 | IS200/IS605 | 1 |
| ISNpu13 | Tn3 | 1 |
| ISBth15 | IS200/IS605 | 2 |
| ***P. forsythia* T98** |  |  |
| ISBs2 | IS630 | 2 |
| ISBcy1 | IS1182 | 4 |
| ISBse2 | IS1182 | 2 |
| ISBce3 | IS200/IS605 | 1 |
| ISRta2 | IS256 | 1 |
| ISArsp6 | Tn3 | 1 |
| ISBsp4 | IS1182 | 1 |
| ISFba3 | IS1182 | 1 |
| ***P. beijingensis* 1-18** |  |  |
| ISTte1 | IS1182 | 2 |
| IS655 | IS3 | 2 |
| ISClsp1 | IS3 | 2 |
| IS655 | IS3 | 1 |
| ISEfa8 | IS3 | 1 |
| ISHaha5 | IS110 | 2 |
| ISDha13 | IS200/IS605 | 1 |
| ISBpl1 | IS5 | 1 |
| IS655 | IS3 | 1 |
| ISClsp1 | IS3 | 1 |
| ISNpu13 | Tn3 | 1 |
| ISBbr1 | IS1182 | 1 |
| ISEf1 | IS256 | 1 |
| ISTte1 | IS1182 | 1 |
| ***P. polymyxa* TD94** |  |  |
| ISBcy1 | IS1182 | 3 |
| IS655 | IS3 | 1 |
| ISClsp1 | IS3 | 1 |
| ISEfa8 | IS3 | 1 |
| ISBth7 | IS1182 | 1 |
| ISLtaq1 | IS3 | 1 |
| ISBth8 | IS3 | 1 |
| ISCef8 | IS5 | 1 |
| ***P. polymyxa* 1-43** |  |  |
| ISBth8 | IS3 | 4 |
| ISBsu1 | IS3 | 3 |
| ISErh1 | IS3 | 1 |
| ***Paenibacillus* sp. 1-49** |  |  |
| ISBse2 | IS1182 | 2 |
| IS655 | IS3 | 1 |
| ISClsp1 | IS3 | 1 |
| ISEfa8 | IS3 | 1 |
| IS1485 | IS3 | 1 |
| IS655 | IS3 | 1 |
| ISClsp1 | IS3 | 1 |
| ISBth10 | IS3 | 1 |
| ISHaha3 | IS3 | 1 |
| ISSmu1 | IS3 | 1 |
| ISDha16 | IS701 | 1 |
| IS642 | IS630 | 1 |
| ISHaha5 | IS110 | 1 |
| ISSmi2 | IS1182 | 1 |
| ISBse2 | IS1182 | 1 |
| ISBcy1 | IS1182 | 1 |
| ISBsp4 | IS1182 | 1 |
| ISCde1 | IS66 | 1 |
| ISBce19 | IS3 | 1 |
| ISBce18 | IS3 | 1 |
| ISBs2 | IS630 | 2 |
| ISBcy1 | IS1182 | 1 |
| ISBse2 | IS1182 | 2 |
| ISBsp4 | IS1182 | 1 |
| ISBbr1 | IS1182 | 1 |
| ISBsp1 | IS1595 | 1 |
| [***P. sabinae***](http://www.ncbi.nlm.nih.gov/nuccore/DQ338444.1) **T27** |  |  |
| ISDha13 | IS200/IS605 | 3 |
| IS200S | IS200/IS605 | 1 |
| ISCth10 | IS200/IS605 | 1 |
| ISSsu4 | IS200/IS605 | 2 |
