# Supplementary material for: Comparative Genomic Analysis of N2-Fixing and Non-N2-Fixing Paenibacillus spp.: Organization, Evolution and Expression of the Nitrogen Fixation Genes
Source: PLoS Genet. 2014 Mar 20;10(3):e1004231. doi: 10.1371/journal.pgen.1004231 (PMC3961195; doi:10.1371/journal.pgen.1004231)
Supplement: Table S4 — Prophages present in the genomes of 31 Paenibacillus strains. The following information is provided for each prophage: insertion site, size, locus tags, and selected cargo genes. (DOCX) [file pgen.1004231.s019.docx]

| **Strains** | **Size (kb)** | **Completeness** | **tRNA No.** | **Total protein** | **GC content** | **Cargo genes** |
| --- | --- | --- | --- | --- | --- | --- |
| *Paenibacillus* sp. JDR 2 |  |  |  |  |  |  |
|  | 14.4 | incomplete | 0 | 17 | 49.2% | - |
|  | 13.2 | incomplete | 1 | 20 | 48.3% | - |
|  | 14 | incomplete | 0 | 20 | 48.33% | phosphoglucomutase |
| *Paenibacillus* sp. Y412MC10 | 41 | incomplete | 0 | 21 | 49% | GCN5-like N-acetyltransferase; periplasmic solute-binding protein; ABC-3 protein |
|  | 31.9 | intact | 0 | 42 | 51.44% | AraC family transcriptional regulator;lysine exporter protein LysE/YggA |
| *P. mucilaginosus* KNP414 | 30.5 | intact | 0 | 29 | 50.86% | - |
| *P. mucilaginosus* K02 | 35.6 | intact | 0 | 41 | 48.12% | amine oxidase |
|  | 17.9 | intact | 0 | 18 | 53.18% | - |
| *P. mucilaginosus* 3016 | 20.2 | intact | 0 | 22 | 53.12% |  |
|  | 7.8 | incomplete | 0 | 11 | 56.35% | TetR family transcriptional regulator; multidrug transporter |
|  | 28.1 | incomplete | 0 | 24 | 50.25% | - |
| [*P. polymyxa* E681](ftp://ftp.ncbi.nih.gov/genomes/Bacteria/Paenibacillus_polymyxa_E681_uid53477/) | 19.4 | questionable | 0 | 21 | 48.43% | - |
|  | 18.6 | questionable | 0 | 29 | 37.81% | NTP pyrophosphohydrolase |
| *P. polymyxa* SC2 | 20.3 | incomplete | 0 | 25 | 46.54% | aminoglycoside 6-adenylyltransferase; |
|  | 12.7 | incomplete | 0 | 17 | 47.84% | aryl alcohol dehydrogenase; oxidoreductase; aryl alcohol dehydrogenase |
|  | 34.7 | questionable | 0 | 14 | 44.4% | - |
| *P. curdlanolyticus* YK9 | 11.6 | incomplete | 0 | 14 | 48.88% | - |
|  | 18.3 | incomplete | 0 | 23 | 53.11% |  |
| *Paenibacillus* sp. HGF5 | 29.7 | incomplete | 0 | 21 | 49.85% | NUDIX hydrolase; ABC-3 protein; periplasmic solute-binding protein; TmrB; GCN5-like N-acetyltransferase; glyoxalase/bleomycin resistance protein/dioxygenase |
|  | 38.7 | intact | 0 | 26 | 47.7% | - |
|  | 33.6 | intact | 0 | 39 | 52.1% | - |
| *Paenibacillus* sp. HGF7 | 29.9 | incomplete |  | 14 | 49.28% | helix-turn-helix domain-containing protein; |
|  | 17.7 | incomplete |  | 17 | 38.08% |  |
|  | 18 | incomplete |  | 24 | 35.98% |  |
|  | 15.1 | incomplete |  | 16 | 56.34% | membrane protein; N-acetyltransferase GCN5 |
| *P. dendritiformis* C454 | 59.1 | questionable | 0 | 40 | 52.33% | HicB family protein; helix-turn-helix domain-containing protein |
|  | 31.9 | intact | 0 | 39 | 55.17% |  |
|  | 58.6 | intact | 1 | 61 | 49.03% | lipolytic protein g-d-s-l family |
|  | 14.9 | incomplete | 0 | 22 | 57.68% | - |
| *P. elgii* B69 | 14.6 | questionable | 0 | 23 | 53.82% | CAAX amino terminal protease; phosphoesterase |
|  | 20.9 | incomplete | 0 | 31 | 33.19% | YolD-like protein |
|  | 17.6 | incomplete | 0 | 30 | 33.56% | - |
|  | 31.1 | incomplete | 0 | 25 | 33.35% | XRE family transcriptional regulator; YapH protein; RNA methyltransferase |
|  | 47.9 | intact | 0 | 71 | 50.93% | transition state transcriptional regulatory protein abrb; Resolvase domain; DNA-binding protein |
|  | 16.3 | incomplete | 0 | 21 | 37.39% | - |
|  | 18 | incomplete | 0 | 23 | 49.18% | transcriptional regulator |
|  | 23.2 | incomplete | 0 | 13 | 44.22% | - |
|  | 31.7 | intact | 0 | 33 | 49.45% | - |
|  | 12.9 | incomplete | 0 | 17 | 49.17% | - |
| *P. lactis*154 | 41.9 | intact | 2 | 58 | 50% | XRE family transcriptional regulator; GCN5-like N-acetyltransferase |
|  | 29.2 | intact | 0 | 37 | 54.11% | - |
|  | 16.2 | incomplete | 0 | 18 | 46.19% | - |
| *P. peoriae* KCTC 3763 | 16.2 | incomplete | 0 | 17 | 39.94% | group-specific protein |
|  | 18.2 | incomplete | 0 | 24 | 48.55% | - |
| *Paenibacillus* sp. oral taxon786 str. D14 | 12.9 | incomplete | 0 | 16 | 51.75% | Mor transcription activator domain-containing protein |
| *P. vortex* V453 | 33.6 | intact | 0 | 44 | 50.71% | lysine exporter protein; AraC family transcriptional regulator |
| *P. polymyxa* WLY78 | 14.3 | incomplete | 0 | 17 | 48.81% | - |
| *P. polymyxa* TD94 | 14.3 | incomplete | 0 | 16 | 44.05% | - |
| *P. polymyxa* 1-43 | 20.3 | incomplete | 0 | 26 | 46.42% | aminoglycoside 6-adenylyltransferase |
| *P. beijingensis* 1-18 | 17.8 | incomplete | 0 | 16 | 44.68% | Site-specific recombinase; |
|  | 15 | incomplete | 0 | 12 | 39.49% | - |
|  | 23.9 | incomplete | 0 | 24 | 42.27% | - |
|  | 20.6 | incomplete | 0 | 25 | 48.64% | aminoglycoside 6-adenylyltransferase |
|  | 5.8 | incomplete | 0 | 9 | 48.22% | Transposase; permease |
|  | 25.8 | questionable | 0 | 31 | 45.06% | - |
|  | 17.1 | incomplete | 0 | 24 | 42.94% | - |
|  | 25.2 | incomplete | 1 | 20 | 45.22% | Non-ribosomal peptide synthetase module containing protein; enoyl-ACP reductase; Trk-type K^+^ transporter membrane component; transcription elongation factor; tRNA-dihydrouridine synthase 1; dihydroneopterinaldolase; aminodeoxychorismatelyase |
| *Paenibacillus* sp. 1-49 | 31.5 | incomplete | 0 | 18 | 19.91% | - |
| *Paenibacillus* sp. Aloe-11 | 15.4 | incomplete | 0 | 30 | 43.59% | - |
|  | 17.6 | incomplete | 0 | 21 | 48.46% | - |
| *P. terrae* HPL-003 | 22 | incomplete | 0 | 27 | 48.5% | - |
|  | 15.9 | Questionable | 0 | 31 | 40.29% | - |
| *P. massiliensis* T7 | 19.2 | incomplete | 0 | 22 | 47.82% | - |
|  | 31.7 | intact | 0 | 36 | 47.76% | CN5 family acetyltransferase |
| *P. graminis* RSA19 | 36.9 | intact | 2 | 26 | 47.08% | - |
|  | 14.6 | incomplete | 0 | 25 | 49.88% | - |
|  | 14.9 | incomplete | 0 | 22 | 48.65% | - |
| *P. sonchi* X19-5 | 19.1 | incomplete | 0 | 27 | 49.96% | response regulatory protein |
| *P. azotofixans* ATCC 35681 | 17.5 | incomplete | 0 | 25 | 50.19% | ABC transporter ATP-binding protein |
|  | 23.5 | incomplete | 0 | 32 | 51.66% | - |
|  | 16.3 | incomplete | 0 | 22 | 49.02% | - |
| *P. sophorae* S27 | 19.5 | incomplete | 0 | 21 | 53.19% | - |
|  | 14.4 | incomplete | 0 | 16 | 50.32% | Glycosyltransferase EpsD; baseplate J family protein |
|  | 8 | incomplete | 0 | 7 | 40.65% | deoxyinosine 3'endonuclease-like protein |
|  | 8 | incomplete | 0 | 13 | 54.00% | - |
|  | 37.3 | incomplete | 0 | 24 | 50.58% | transcriptional regulator |
|  | 16.4 | incomplete | 0 | 22 | 51.71% | - |
|  | 42 | incomplete | 0 | 21 | 33.06% | DNA/RNA helicase |
|  | 37 | incomplete | 0 | 25 | 33.97% | - |
|  | 36 | incomplete | 0 | 30 | 35.26% | - |
| *P. zanthoxyli* JH29 | 14.6 | incomplete | 0 | 21 | 51.96% | - |
| *P. forsythia* T98 | 17.9 | incomplete | 0 | 27 | 51.02% | Rhs family protein; holin |
|  | 9.3 | incomplete | 0 | 16 | 55.96% | - |
|  | 19.6 | incomplete | 0 | 26 | 50.89% | DNA replication protein DnaD; transcriptional regulator; XRE family transcriptional regulator |
| [*P. sabinae*](http://www.ncbi.nlm.nih.gov/nuccore/DQ338444.1) T27 | 27.7 | incomplete | 0 | 40 | 45.73% | XRE family transcriptional regulator |
