# Supplementary material for: Comparative Genomic Analysis of N2-Fixing and Non-N2-Fixing Paenibacillus spp.: Organization, Evolution and Expression of the Nitrogen Fixation Genes
Source: PLoS Genet. 2014 Mar 20;10(3):e1004231. doi: 10.1371/journal.pgen.1004231 (PMC3961195; doi:10.1371/journal.pgen.1004231)
Supplement: Table S5 — The nitrogen fixation genes and nitrogenase-like genes in the nitrogen-fixing Paenibacillus strains. (DOCX) [file pgen.1004231.s020.docx]

| Strains | Nitrogen-fixing genes  The *nif* cluster Other *nif* genes | *vnf*/*anf* genes | Nitrogenae-like genes |
| --- | --- | --- | --- |
| *P. terrae* HPL-003 | *nifBHDKENXhesAnifV* |  |  |
| *Paenibacillus* sp. Aloe-11 | *nifBHDKENXhesAnifV* |  |  |
| *P. graminis* RSA19 | *nifBHDKENXhesAnifV* |  |  |
| *P. sonchi* X19-5 | *nifBHDKENXhesAnifV* |  |  |
| *Paenibacillus* sp. WLY78 | *nifBHDKENXhesAnifV* |  |  |
| *P. massiliensis* T7 | *nifBHDKENXhesAnifV* |  |  |
| *P. beijingensis* 1-18 | *nifBHDKENXhesAnifV* |  |  |
| *Paenibacillus* sp. TD94 | *nifBHDKENXhesAnifV* |  |  |
| *Paenibacillus* sp. 1-43 | *nifBHDKENXhesAnifV* |  |  |
| *Paenibacillus* sp. 1-49 | *nifBHDKENXhesAnifV* |  |  |
| *P. sabinae* T27 | *nifBHDKENXhesAnifV* 2 *nifB,* 2 *nifH, nifE, nifN* |  | 2 *nifH-*like, 5 *nifDK-*like |
| *P. azotofixans* ATCC 35681 | *NifBHDKENXhesAnifV nifB, nifE, nifN, nifX,* 2 *nifV* | *vnfHDGKEN* | 2 *nifH-*like, 4 *nifDK-*like |
| *P. sophorae* S27 | *nifBHDKENXhesAnifV nifE, nifN, nifX, nifV* | *anfHDGK* | 1 *nifH-*like, 4 *nifDK-*like |
| *P. zanthoxyli* JH29 | *nifBHDKENXhesAnifV*  *nifB, nifE, nifN, nifX,* 2 *nifV* | *vnfHDGKEN* | 1 *nifH-*like, 4 *nifDK-*like |
| *P. forsythia* T98 | *nifBHDKENXhesAnifV nifB, nifE, nifN, nifX* | *anfHDGK* | 2 *nifH-*like, 6 *nifDK-*like |
